# Efficacy and safety of different inhaler types for asthma and chronic obstructive pulmonary disease. a systematic review and meta-analysis

**DOI:** 10.1038/s41533-026-00488-4

**Published:** 2026-02-16

**Authors:** Michael J. Loftus, Miranda S. Cumpston, Shannon Barnes, John Blakey, Allan Glanville, Steve McDonald, Loyal Pattuwage, Megan Rees, Rachel Silk, Heath White, Tari Turner, Karin Leder

**Affiliations:** 1 Planetary Health Division, School of Public Health and Preventive Medicine, Monash University, Melbourne, Australia; 2 Health and Climate Initiative, Faculty of Medicine, Nursing and Health Sciences, Monash University, Melbourne, Australia; 3 Australian Living Evidence Collaboration, School of Public Health and Preventive Medicine, Monash University, Melbourne, Australia; 4 Respiratory Department, Sir Charles Gairdner Hospital, Perth, Australia; 5 Curtin Medical School, Faculty of Health Sciences, Curtin University, Perth, Australia; 6 Macquarie Respiratory and Sleep Unit, Macquarie University, Sydney, Australia; 7 Department of Respiratory and Sleep Disorders Medicine, The Royal Melbourne Hospital, Melbourne, Australia; 8 Department of Medicine, Royal Melbourne Hospital, University of Melbourne, Melbourne, Australia

**Keywords:** Outcomes research, Asthma, Chronic obstructive pulmonary disease, Adverse effects

## Abstract

Pressurised metered dose inhalers (pMDIs) contain propellant gases with high global warming potential yet remain a cornerstone of management for asthma and chronic obstructive pulmonary disease (COPD). The aim of this study was to determine whether non-propellant alternatives of dry powder inhalers (DPIs) and soft mist inhalers (SMIs) had similar efficacy and safety. A systematic review was performed finding 44 randomised trials (24,710 participants) and moderate certainty evidence for most outcomes. No statistically significant or clinically important differences were found between inhaler types for any assessed measure. For asthma maintenance, the mean difference in peak expiratory flow rate between groups was 1.07 L/min (95% confidence interval [CI] -0.93 to 3.06). For COPD, the mean difference in FEV_1_ between groups was 0.01 L (95% CI -0.01 to 0.02). While the choice of optimal inhaler for an individual patient is a multifaceted decision, this review provides reassurance that non-pMDI devices can perform equally well.

## Introduction

Asthma and chronic obstructive pulmonary disease (COPD) are highly prevalent globally, affecting hundreds of millions of individuals^1,2^. A cornerstone of therapy for these conditions is the directed delivery of effective medications via inhalers^3,4^. Pressurised metered dose inhalers (pMDIs) are one of the most frequently used inhaler devices, with almost one billion manufactured every year^5^. Current pMDIs contain fluorinated propellant gases (F-gases) which are potent greenhouse gases – they exert an effect thousands of times greater than the equivalent volume of carbon dioxide (CO_2_). For example, a single inhaler of a common short-acting beta-agonist product, using the propellant HFA-134a, has the same global warming potential as 25.2 kg CO_2_ (equivalent to driving over 120 kilometres in a petrol car)^6^. The healthcare sector contributes 4-5% of global greenhouse emissions^7,8^, and the proportionally large contribution of inhalers (particularly pMDIs) is increasingly recognised. In the United Kingdom, pMDIs alone are responsible for around 3% of the entire National Health Service carbon footprint^9^.

A key component of this issue is the marked variation between countries in the proportion of prescribed inhalers that are pMDIs, due to both cultural and cost reasons. For example, there is a seven-fold difference in pMDI prescribing rates across northern European countries^10^. These observations in the face of the climate crisis have led to a call for much greater use of propellant-free options such as dry powder inhalers (DPIs) and soft mist inhalers (SMIs), especially in countries that predominantly use pMDIs^11^. A conscious consideration of the environmental impact is now included in inhaler guidelines in the UK^12^.

However, some uncertainty remains as to whether there is equipoise between the benefit of pMDIs and propellant-free inhalers for management of asthma and COPD. Previous systematic reviews with a narrow focus have considered this question for different drug classes across both conditions; however, these reviews are now 20 or more years old and did not include many drugs, formulations (e.g. extrafine particles) and devices (e.g. SMIs) that are now used routinely^13–16^. Some countries have made calls to move away from pMDIs when clinically safe to do so^17^, however strong contemporary data are needed to support such decisions. We therefore undertook a comprehensive systematic review and meta-analysis to establish if there was any evidence of a difference in clinical effectiveness or safety of treating individuals with asthma or COPD acutely or for maintenance therapy with pMDIs versus propellant-free devices, when the drugs and doses administered were broadly equivalent.

## Methods

This review was not registered, but we developed a protocol before commencing the review, containing additional detail on the methods used and a complete list of changes made to the planned methods^18^. This review is reported in accordance with the Preferred Reporting Items for Systematic Reviews and Meta-Analyses statement^19^.

### Search strategy

We searched PubMed, Embase and the Cochrane Central Register of Controlled Trials to 25 September 2025, as well as reference lists of systematic reviews identified in the search. Full search strategies are provided in the Supplementary Information (Section A, Tables S1-S3).

### Eligibility criteria

#### Study designs

Only randomised controlled trials (RCTs) were eligible for inclusion. In accordance with the protocol, observational studies were initially identified in the search but not included in the review as sufficient RCTs were available. We excluded studies not written in English and crossover studies (due to insufficient data regarding washout periods in many studies).

#### Population

We included studies of patients of any age with confirmed asthma or COPD. Asthma studies were categorised as management of acute asthma episodes (e.g. presenting for emergency care) or asthma maintenance therapy. Results were analysed separately for these three conditions. There were no studies comparing relevant devices in acute COPD.

#### Intervention

We included studies comparing the delivery of equivalent inhaled medication(s) by either DPI or SMI (grouped together as non-pMDI inhalers) versus pMDI. Studies of nebulised medication delivery were not included. Medications were considered ‘equivalent’ if they were from the same drug class (e.g. short-acting beta-agonist (SABA), or inhaled corticosteroid (ICS)) and given at approximately equipotent doses. ICS doses were categorised into ‘high,’ ‘medium’ and ‘low’ dose according to National Institute for Health and Care Excellence (NICE) guidelines^20^. Non-ICS medication doses were classified as ‘standard’ based on comparative clinical therapeutic equivalence, and ‘high’ if a multiple of that standard dose was used^3,4^. Studies assessing different combinations of drug classes (e.g. ICS-alone versus ICS/SABA combination) were excluded. We permitted slight variations in the frequency of medication administration (e.g. one versus two doses per day) but excluded larger discrepancies (e.g. once versus three or four doses per day) due possible differences in adherence, pharmacokinetic profiles and therefore effect. There were no restrictions on which drugs participants were taking prior to the trial, or on lead-in or washout periods. Results were analysed together for all drugs and doses.

The inclusion criteria for interventions and the definition of equipotency were intentionally broad to include the most complete picture possible of the available evidence comparing treatment regimens with and without propellant gases. Differences in effects (heterogeneity) observed in the results could then be fully investigated through subgroup analysis.

#### Outcomes

Our primary outcomes were physiological lung function measurements (forced expiratory volume in 1 s (FEV_1_) and peak expiratory flow rate (PEFR)), symptom control (any scale), quality of life (any scale), exacerbations (as defined by each study) and use of additional reliever medication (any measure). Secondary outcomes focused on safety, including mortality, overall or treatment-related adverse events (AEs) and serious adverse events (SAEs) as defined by each study. Studies were eligible if they reported one or more primary or safety outcomes.

We had initially planned to report hospital admissions and emergency room attendance separately, but these were frequently incorporated by our included studies into composite measures of adverse events or exacerbations, and so we have reported these composite outcomes in this review. We had also planned to include additional secondary measures of satisfaction, adherence to therapy and inhaler technique, but found that many studies measuring these factors did not address effectiveness or safety outcomes and were therefore ineligible for inclusion in the review. To avoid presenting misleading estimates of these outcomes from a subset of the available literature, we decided to exclude these outcomes.

Studies were excluded if the duration of follow-up after commencement of treatment was less than 48 h for COPD and asthma maintenance, but there was no minimum duration of treatment or follow-up for studies of acute asthma exacerbations.

Minimal clinically important differences (MCIDs) for various outcomes in asthma and COPD were identified from the published literature where possible or otherwise determined by consensus of our Respiratory Experts; these are summarised in the Supplementary Information (Section B, Table S4).

### Study selection and data extraction

Title and abstract screening and full-text review were conducted using Covidence software^21^. All identified titles were initially screened by one of MJL, LP or HW, before all abstracts of papers deemed potentially relevant were independently screened by two authors (MJL and LP). These two authors then performed independent full-text reviews of all remaining papers. Any disputes were resolved by consensus, or by consultation with the review content experts (AG, JB, MR) where needed.

We extracted data including the study design, clinical condition of participants, inclusion of children and/or adults, inhaler type, drugs and doses used, particle size (normal or extra fine), use of spacers, and outcome data. Using a standardised extraction form, one researcher (SB, MJL, LP or RS) extracted and a second researcher verified the data, with inconsistencies discussed until consensus was reached. To improve consistency of data extracted from figures, a web-based plot digitising software was used^22^. No additional data were sought from authors of included studies. Where studies reported results at more than one time point, the latest time point was used. Within each analysis, only one measure from each study was included. Different measures of the same outcome and measures taken at different time points from different studies were combined in the analysis where possible.

### Risk of bias

Studies were assessed for risk of bias using the Cochrane Risk of Bias tool^23^. Assessment was completed independently for all included studies by two of SB, LP and RS, and reviewed by a third author (MC). Studies assessed as high risk for any domain were assessed overall as being at high risk of bias. Studies assessed as unclear risk for three or more domains, with no high-risk domains, were assessed overall as being at unclear risk of bias. Studies assessed as unclear risk for two or fewer domains, with no high-risk domains, were assessed overall as being at low risk of bias.

### Data analysis

#### Data synthesis

Included studies were grouped by condition (asthma maintenance, asthma acute exacerbations and COPD) for analysis and the available outcome measures were tabulated for selection as described above. Where two or more studies within a group reported results for an outcome of interest, we performed meta-analysis using a random-effects model and inverse variance statistical methods using RevMan Web^24^. Where possible, we reported dichotomous outcomes as risk ratios (RRs) with 95% confidence intervals, and continuous outcomes as mean differences (where all studies used the same outcome measure), or standardised mean differences (where studies used a variety of outcome measures), with 95% confidence intervals.

Where additional studies reported outcome measures or data that could not be included in the meta-analysis, effect estimates were calculated where possible and all results were reported in table format. No synthesis was performed on these results.

#### Imputation and standardisation

Where results were reported with no measure of variance, we imputed standard deviations (SDs) for group-level data from other studies that used the same outcome measure, using the average of SDs from the intervention and control groups of the study with the highest available SD values for the same measure, as a conservative approach. Sensitivity analysis was conducted excluding studies with imputed data from the analysis, and in all cases no meaningful change on the overall meta-analysis results was observed. For standardised analyses, where studies used a range of outcome measures with opposing directions of effect (e.g. scales for which a lower score indicated improvement, and scales for which a higher score indicated improvement), results for the least frequent direction were multiplied by -1 in the analysis. Also, for standardised analyses, either change or endpoint scores were used for all included studies, based on which was the most frequently reported for that outcome. Where outcome data was converted from endpoint to change scores, appropriate measures of variance were also calculated where possible, or otherwise imputed as described above.

#### Heterogeneity and subgroup analyses

Heterogeneity was assessed using the I^2^ statistic. Exploration of the causes of heterogeneity was planned using subgroup analyses, including adults and adolescents (13+ years) vs children (up to 12 years), and SMI vs DPI inhalers. An additional subgroup analysis was conducted to explore the influence of study funding by the manufacturer of either the pMDI or non-pMDI inhalers, or neither. These subgroup analyses were conducted using the primary outcome of FEV_1_, which was commonly measured across the greatest number of studies.

#### Certainty in the evidence

The possibility of reporting bias was considered in the context of the available studies. The presence of small study effects that may indicate reporting bias was assessed using funnel plots where 10 or more studies contributed to a meta-analysis.

We assessed certainty in the evidence using the Grading of Recommendations, Assessment, Development, and Evaluations (GRADE) approach^25^, incorporating the risk of bias, imprecision, inconsistency, indirectness and reporting bias. We summarised assessments for main outcomes in ‘summary of findings’ tables and used standardised language to report the certainty of results^26^.

## Results

### Description of studies

**Fig. 1:**
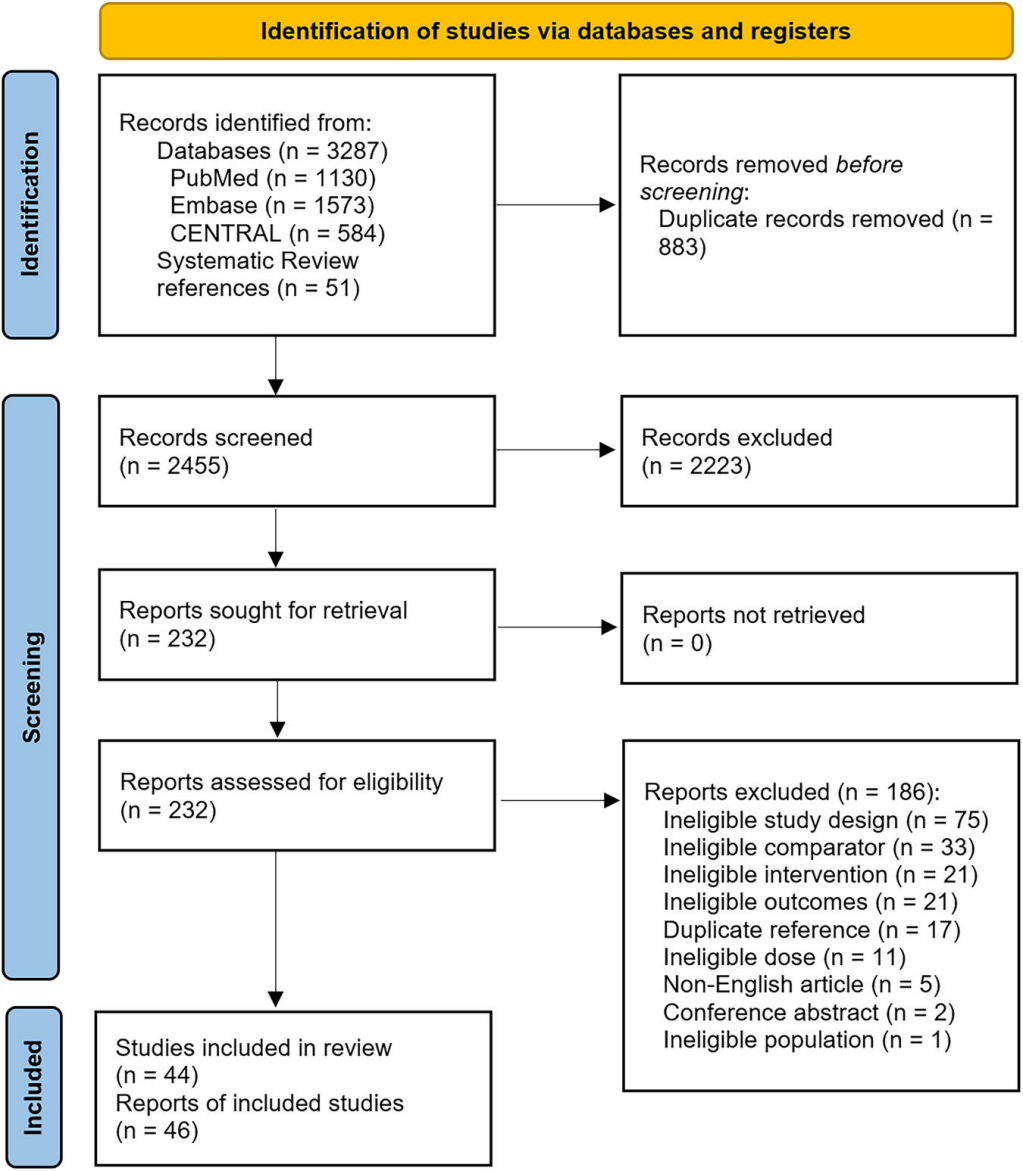
Flow diagram of the systematic review following the Preferred Reporting Items for Systematic Reviews and Meta-Analyses guidelines.

Of 3287 records identified in the search and 51 records identified from references of systematic reviews, 44 RCTs (reported in 46 papers) were included (32 on asthma maintenance therapy, five on acute asthma exacerbations, and seven on COPD) (Fig. 1 and Table 1). All studies of acute asthma exacerbations were in children. The majority of studies (38/44, 86.4%) were funded by device manufacturers, including all seven studies assessing patients with COPD (Table 1). Among these 38 studies, in 23 (60.5%) the study received funding from the manufacturer of the DPI/SMI device, in 10 (26.3%) the study received funding from the manufacturer of the pMDI device, and in 5 (13.2%) the same manufacturer produced the devices used in both study arms and funded the study (Table S5 in the Supplementary Information).

**Table 1:** Characteristics of included studies.

Publication	Country	Patient ages	pMDI arm	Comparator arm^a^	Max. duration of treatment	Funded by device manufacturer	Overall risk of bias
***Asthma – Maintenance Therapy***							
Amar, 2017^60^^,^^61^	Multinational (18 countries)	5 to 11 years	MF 50 µg bd (n = 120)	MF 100 µg od (n = 125)	12 weeks	Yes	High
Barnes, 2013^62^	Multinational (4 countries)	18 to 65 years	Extrafine BDP/FOR 200/12 µg bd (n = 215)	FP/SAL 250/50 mg bd (n = 216)	12 weeks	Yes	Unclear
Bateman, 2001^63^	Multinational (10 countries)	12 or more years	FP/SAL 100/50 µg bd (n = 165)	FP/SAL 100/50 µg bd (n = 167)	12 weeks	Yes	Unclear
Bernstein, 2011^64^	Multinational (Unspecified)	12 or more years	MF/FOR 200/10 µg bd (n = 371)	FP/SAL 250/50 µg bd (n = 351)	12 weeks	Yes	High
Bodzenta-Lukaszyk, 2012^65^	Multinational (5 countries)	12 or more years	FP/FOR 250/10 µg bd (n = 140)	BUD/FOR 400/12 µg bd (n = 139)	12 weeks	Yes	Low
Bracamonte, 2005^66^	Multinational (11 countries)	4 to 11 years	FP/SAL 100/50 µg bd (n = 215)	FP/SAL 100/50 µg bd (n = 213)	12 weeks	Yes	Low
Bronsky, 1987^67^	USA	12 or more years	ALB 180 µg qid (n = 116)	ALB 200 µg qid (n = 115)	12 weeks	Yes	High
Busse, 2008 / O’Connor, 2010^68,69^	USA	12 or more years	BUD/FOR 320/9 µg bd (n = 427 [Busse]; n = 368 [O’Connor])	FP/SAL 250/50 µg bd (n = 406 [Busse]; n = 350 [O’Connor])	30 weeks	Yes	High
Dusser, 2005^70^	Multinational (8 countries)	18 to 70 years	FOR 12 µg bd (n = 225)	FOR 12 µg bd (n = 220)	12 weeks	Yes	Unclear
Kanniess, 2015^71^	Multinational (7 countries)	18 or more years	Extrafine BDP/FOR 100/6 µg bd (n = 251)	Extrafine BDP/FOR 100/6 µg bd (n = 251)	8 weeks	Yes	Low
Kemp, 1989^72^	USA	4 to 12 years	ALB 180 µg qid (n = 104)	ALB 200 µg qid (n = 100)	12 weeks	Unclear	Low
Koskela, 2000^73^	Finland	pMDI mean 45 (SD 15) years; DPI mean 41 (SD 17) years	BDP 400 µg bd (n = 76)	BDP 400 µg bd (n = 68)	8 weeks	Yes	Unclear
Lundback, 1993^74^	Multinational (10 countries)	15 to 91 years	FP 250 µg bd (n = 193)	FP 250 µg bd (n = 198)	6 weeks	Yes	Unclear
Lundback, 1994^75^	Multinational (10 countries)	17 to 76 years	FP 100 µg bd (n = 146)	FP 100 µg bd (n = 150)	4 weeks	Yes	Unclear
Morice, 2007^76^	Multinational (8 countries)	12 or more years	BUD/FOR 320/9 µg bd (n = 234)	BUD/FOR 320/9 µg bd (n = 229)	12 weeks	Yes	Low
Morice, 2008^77^	Multinational (6 countries)	12 or more years	BUD/FOR 320/9 µg bd (n = 446)	BUD/FOR 320/9 µg bd (n = 227)	52 weeks	Yes	High
Nelson, 1999^78^	USA	12 or more years	ALB 180 µg qid (n = 92)	ALB 216 µg qid (n = 97)	12 weeks	Yes	Unclear
Papi, 2007^79^	Multinational (13 centres in Europe)	18 to 65 years	BDP/FOR 200/12 µg bd (n = 107)	BUD/FOR 400/12.0 µg bd (n = 109)	12 weeks	Yes	Unclear
Papi, 2012^80^	Multinational (67 Respiratory Clinics in Europe)	18 to 65 years	Extrafine BDP/FOR 200/12 µg bd (n = 206)	FP/SAL 250/50 µg bd (n = 216)	24 weeks	Yes	High
Pauwels, 1996^81^	Multinational (7 countries)	17 or more years	BDP and/or TS at previous doses (n = 506)	BUD and/or TS at doses equivalent to previous use (n = 498)	52 + /- 4 weeks	Yes	High
Poukkula, 1998^82^	Finland	18 or more years	BDP 500 µg bd (n = 74)	BDP 500 µg bd (n = 74)	12 weeks	Yes	High
Reichel, 2001^83^	Multinational (6 countries)	18 to 75 years	BDP 200 µg bd (n = 98)	BUD 400 µg bd (n = 95)	6 weeks	Yes	High
Srichana, 2016^84^	Thailand	18 to 60 years	BUD 200 µg bd (n = 18)	BUD 200 µg bd (n = 18)	12 weeks	No	High
Stradling, 2000^85^	UK	18 or more years	BDP < 2 mg/day (n = 106)	BDP < 2 mg/day (n = 98)	12 weeks	Yes	Unclear
Van Noord, 2001^86^	Multinational (13 countries)	12 to 82 years	FP/SAL 500/50 µg bd (n = 176)	FP/SAL 500/50 µg bd (n = 161)	12 weeks	Yes	Unclear
Vincken, 2004^87^	Multinational (3 countries)	18 to 65 years	IB/FEN 40/100 µg qid (n = 159)	IB/FEN 20/50 µg qid (SMI) (n = 161)	12 weeks	Yes	High
Von Berg, 2004^88^	Multinational (4 countries)	6 to 15 years	IB/FEN 40/100 µg tid (n = 177)	IB/FEN 20/50 µg tid (SMI) (n = 180)	4 weeks	Yes	High
Von Berg, 2007^89^	Multinational (8 countries)	6 to 11 years	CIC 160 µg od (n = 416)	BUD 400 µg od (n = 205)	12 weeks	Yes	Unclear
Wardlaw, 2004^90^	–Multinational (European and Canadian sites)	12 or more years	FP 250 µg bd (n = 85)	MF 400 µg od (n = 82)	8 weeks	Yes	High
Wolfe, 2000^91^	USA	12 or more years	SAL 42 µg bd (n = 166)	SAL 50 µg bd (n = 165)	12 weeks	Yes	Unclear
Zheng, 2023^92^	China	18 or more years	Extrafine BDP/FOR 200/12 µg bd (n = 242)	Extrafine BDF/FOR 200/12 µg bd (n = 251)	12 weeks	Yes	Unclear
Zhou, 2025^93^	China	18 or more years	BUD 200 µg bd (n = 78)	BUD 200 µg bd (n = 78)	4 weeks	No	Low
***Asthma – Acute Exacerbation***							
Direkwatanachai, 2011^94^	Thailand	5 to 18 years	Salbutamol 600 µg (n = 68)	Salbutamol 600 µg (n = 71)	60 min	Yes	High
Drblik, 2003^95^	Canada	6 to 16 years	TS 0.5 mg/10 kg (max 2 mg) two doses 30 mins apart (n = 55)	TS 0.5 mg/10 kg (max 2 mg) two doses 30 mins apart (n = 57)	60 min	Yes	Unclear
Khaled, 2014^96^	Bangladesh	6 to 15 years	Salbutamol 400 µg (n = 53)	Salbutamol 400 µg (n = 53)	30 min	No	High
Lodha, 2004^97^	India	5 to 15 years	Salbutamol 400 µg (n = 78)	Salbutamol 400 µg (n = 75)	30 min	No	High
Vangveeravong, 2008^98^	Thailand	5 to 18 years	Salbutamol 600 µg (n = 18)	Salbutamol 600 µg (n = 18)	60 min	No	High
***Chronic Obstructive Pulmonary Disease (COPD)***							
Ferguson, 2013^99^	USA	40 or more years	IB/salbutamol 36/206 µg qid [equivalent to 180 µg salbutamol base] (n = 154)	IB/salbutamol 20/100 µg qid (SMI) (n = 157)	48 weeks	Yes	High
Ferguson, 2018^100^	Multinational (7 countries)	40 to 80 years	BUD/FOR 320/10 µg bd (n = 655)	BUD/FOR 400/12 µg bd (n = 219)	24 weeks	Yes	High
Kilfeather, 2004^101^	Multinational (3 countries)	40 or more years	IB/FEN 40/100 qid (n = 220)	IB/FEN 20/50 qid (SMI) (n = 224)	12 weeks	Yes	High
Koser, 2010^102^	USA	40 or more years	FP/SAL 230/42 µg bd (n = 121)	FP/SAL 250/50 µg bd (n = 126)	12 weeks	Yes	Unclear
Maltais, 2019^103^	Multinational (7 countries)	40 to 95 years	Glycopyrrolate/FOR 18/9.6 µg bd (n = 552)	Umeclidinium/VI 62.5/25.0 µg od (n = 552)	24 weeks	Yes	Unclear
Wang, 2020^104^	China	40 to 80 years	BUD/FOR 320/9.6 µg bd (n = 72)	BUD/FOR 400/12 µg bd (n = 72)	24 weeks	Yes	High
Zuwallack, 2010^105^	Multinational (13 countries)	40 or more years	IB/ALB 36/206 µg qid (n = 491)	IB/ALB 20/100 µg qid (SMI) (n = 486)	12 weeks	Yes	Unclear

^a^The comparator is Dry Powder Inhaler (DPI) unless otherwise stated.

bd: twice daily, *BDP* beclomethasone dipropionate, *BUD* budesonide, *CIC* Ciclesonide, *DPI* dry powder inhaler, *FEN* fenoterol hydrobromide, *FF* fluticasone furoate, *FOR* formoterol, *FP* fluticasone propionate, *IB* ipratropium bromide, *MF* mometasone furoate, *od* once daily, *pMDI* pressurised metered dose inhaler, *qid* four times daily, *SAL* salmeterol, *SMI* soft mist inhaler, *tid* three times daily, *TS* terbutaline sulphate, *VI* vilanterol.

Risk of bias assessments are summarised in Figure S1 in the Supplementary Information. Across all 44 studies, 21 were considered high-risk for bias, 6 were low-risk and the remaining 17 were unclear. Of those studies rated at high risk, the most frequent domain assessed as high risk of bias related to blinding of participants and personnel.

### Analyses

Therapy via either a pMDI or non-pMDI device had similar effects on all outcomes of interest. Most evidence was considered moderate certainty, with certainty downgraded due to the proportion of studies at high or unclear risk of bias, but the observed results were highly robust to all sensitivity analyses, and imprecision and heterogeneity were both very low across most outcomes. No indication of reporting bias was identified, and small study effects were not detected in funnel plots for any meta-analyses. Summary of findings tables including GRADE assessments of the certainty of the evidence are presented in the Supplementary Information (Section C, Tables S6-S8).

#### Forced expiratory volume in 1 s (FEV_1_)

**Fig. 2:**
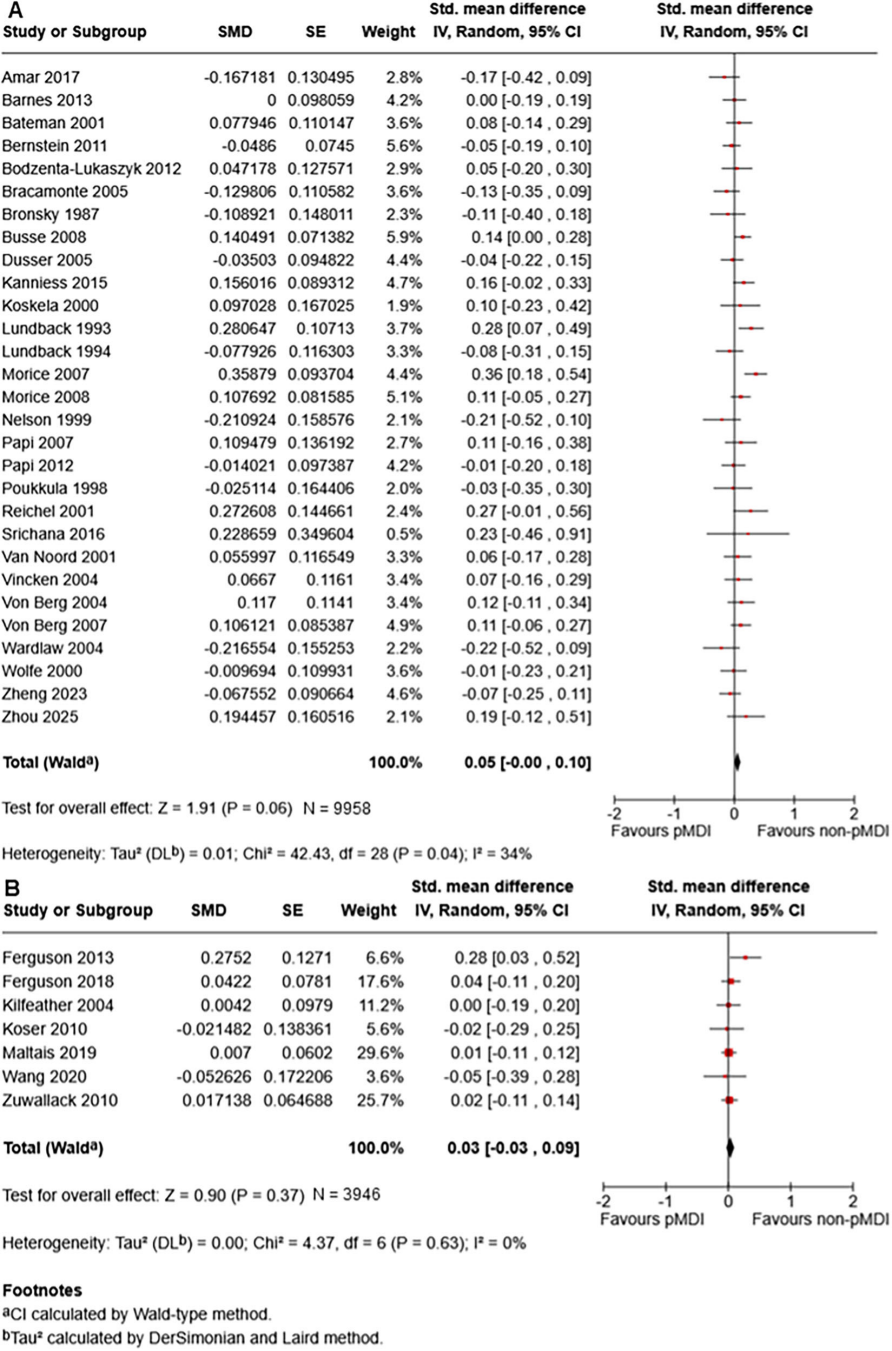
Meta-analysis of the association between device type and forced expiratory volume in 1 s (FEV_1_) in (**A**) asthma maintenance and (**B**) chronic obstructive pulmonary disease. The centre of the squares or diamonds indicates the point estimate and the width is the 95% confidence interval (CI). SE = standard error. SMD = standard mean difference. IV = inverse variance.

There was moderate certainty evidence of little or no difference in FEV_1_ between pMDI and non-pMDI devices for both asthma maintenance (standardised mean difference (SMD) 0.05, 95% CI 0 to 0.10; 29 studies; n = 9958) and COPD (SMD 0.03, 95% CI -0.03 to 0.09; seven studies; n = 3946) (Fig. 2). In asthma maintenance, this equates to a mean difference in percent predicted FEV_1_ of 0.71% (95% CI 0% to 1.42%), well below the MCID of 12%^27^ (see minimal clinically important differences in Table S4 in the Supplementary Information). The effect estimate among COPD patients is equivalent to a mean difference of 0.01 L (95% CI -0.01 L to 0.02 L), well below the MCID of 0.1 L^28^.

Only one study assessed FEV_1_ in acute asthma, which showed low certainty evidence of little to no difference in this population (mean difference 2% predicted, 95% CI -2.9% to 6.9%). One additional study reported FEV_1_ in asthma maintenance that could not be included in the meta-analysis (Table S9 in the Supplementary Information).

#### Peak expiratory flow rate (PEFR)

**Fig. 3:**
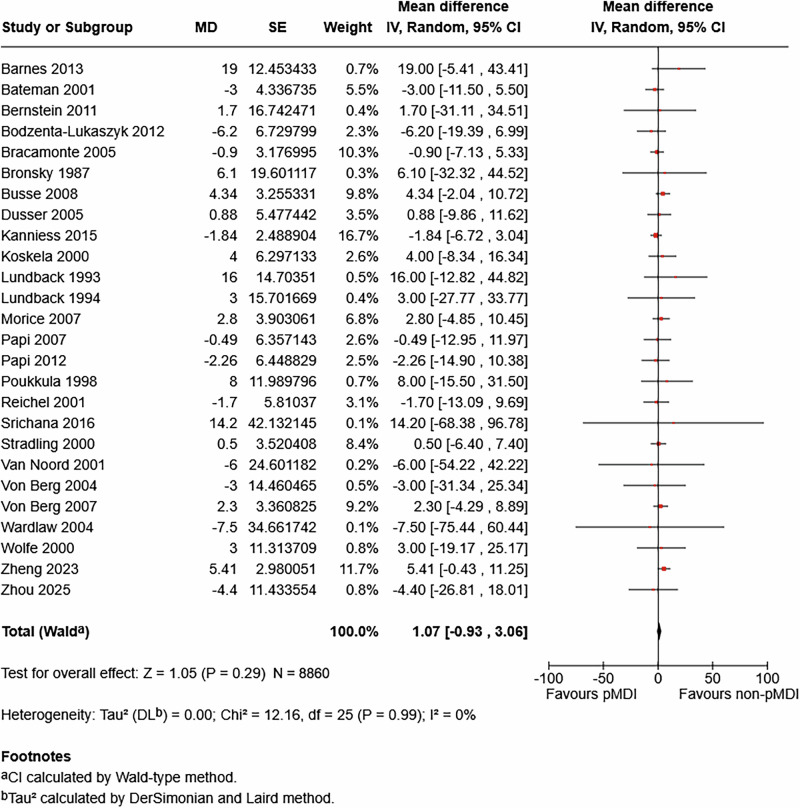
Meta-analysis of the association between device type and peak expiratory flow rate (PEFR) in asthma maintenance. The centre of the squares or diamond indicates the point estimate and the width is the 95% confidence interval (CI). SE = standard error. MD = mean difference. IV = inverse variance.

There was moderate certainty evidence of little or no difference in PEFR between pMDI and non-pMDI devices for asthma maintenance (mean difference 1.07 L/min, 95% CI -0.93 to 3.06; 26 studies; n = 8860) (Fig. 3), with these values well short of the MCID of 18.8 L/min for asthma maintenance studies^29^.

There was low certainty evidence of little or no difference in PEFR between device types for acute asthma exacerbations (mean difference 1.38 L/min, 95% CI -17.97 to 20.73; two studies; n = 259). This difference is smaller than the MCID used of 12% predicted among adults with acute asthma exacerbations^30^ (which equates to approximately 25 L/min in paediatric populations, such as those in the two acute asthma studies included in our analysis). There was moderate certainty evidence of little or no difference among COPD patients (mean difference -2.56 L/min, 95% CI -9.17 to 4.05; two studies; n = 644) (Figure S3 in the Supplementary Information), acknowledging that there is no published MCID for PEFR in COPD.

There was one additional study that measured PEFR in asthma maintenance that could not be included in the meta-analysis (Table S10 in the Supplementary Information).

#### Use of reliever medication

There was moderate certainty evidence of little or no difference in use of reliever medication between pMDI and non-pMDI devices for both asthma maintenance (SMD 0.02, 95% CI -0.06 to 0.09; 13 studies; n = 4308) and COPD (mean difference -0.21 puffs/day, 95% CI = -0.51 to 0.10; three studies; n = 1265) (Figure S4 in the Supplementary Information). The asthma maintenance SMD of 0.02 equates to approximately 0.05 puffs/day (95% CI -0.16 to 0.23), well below the minimal patient perceivable improvement value of 0.81 puffs/day^29^.

There were seven additional studies that reported results on reliever use for asthma maintenance that could not be included in the meta-analysis (Table S11 in the Supplementary Information). This was not a relevant outcome for people with acute asthma exacerbations.

#### Symptom control

Regarding symptom control scores, for asthma maintenance there was moderate certainty evidence of little or no difference between pMDI and non-pMDI devices (SMD -0.04, 95% CI -0.11 to 0.02; eight studies; n = 3836). This SMD of -0.04 equates to approximately -0.02 points (95% CI -0.05 to 0.01) on the Asthma Control Questionnaire (ACQ-7), well below the MCID of 0.5 points. For COPD, using the COPD Assessment Test (CAT) score, there was moderate certainty evidence of little or no difference between pMDI and non-pMDI devices (mean difference -0.59 points, 95% CI = -1.19 to 0.01, one study; n = 1006). The observed mean difference was below the MCID of 2 points (Figure S5 in the Supplementary Information).

For acute asthma exacerbations, using the Modified Wood Clinical Asthma Score there was low certainty evidence of little or no difference between pMDI and non-pMDI devices (mean difference -0.1 points, 95% CI -0.72 to 0.52, one study; n = 32) (Figure S5 in the Supplementary Information).

There were eight additional studies for asthma maintenance and one additional study for acute asthma exacerbations that reported symptom control results that could not be included in the meta-analysis (Table S12 in the Supplementary Information).

#### Quality of life

Comparisons between studies were challenging due to the variety of quality of life scores used across studies. Meta-analysis was only possible for two asthma maintenance studies assessing the proportion of participants with an Asthma Quality of Life Questionnaire score improving ≥0.5 points. There was very low certainty evidence on the effect of device type on quality of life (relative risk (RR) 1.02, 95% CI 0.91 to 1.14; two studies; n = 871) (Figure S6 in the Supplementary Information). Seven further studies reported on quality of life measures (five for asthma maintenance, two for COPD) (Table S13 in the Supplementary Information).

#### Exacerbations

There was moderate certainty evidence of little or no difference between pMDI and non-pMDI devices in the risk of experiencing ≥1 exacerbation for both asthma maintenance (RR 0.87, 95% CI 0.72 to 1.05; 19 studies; n = 7532) and COPD (RR 1.08, 95% CI 0.94 to 1.24; seven studies; n = 4101) (Figure S7 in the Supplementary Information). This was not a relevant outcome for people with acute asthma exacerbations.

#### Adverse events

Results around AEs and SAEs – including those that were deemed to be treatment-related – are summarised in the Supplementary Information (Figures S8-S11). No clinically important difference was demonstrated between pMDI and non-pMDI devices for any condition. It should be noted that for severe and treatment-related adverse events, fewer studies were included in the analysis and fewer events reported, increasing the imprecision of the estimates and reducing the certainty in the estimate.

#### Mortality

No deaths were reported in any studies of asthma maintenance or acute asthma. For COPD, there was low certainty evidence of little or no difference between pMDI and non-pMDI devices (RR 1.40, 95% CI 0.59 to 3.32; six studies; n = 3657) (Figure S12 in the Supplementary Information).

#### Subgroup analyses

Subgroup analyses did not identify any factors that significantly modified the results, as little or no heterogeneity was observed. Studies in either children or adults were available for subgroup analysis by age group in asthma maintenance (Figure S13 in the Supplementary Information, test for subgroup difference P = 0.68). Studies using either SMI or DPI were available for subgroup analysis by non-pMDI device type in COPD (Figure S14 in the Supplementary Information, test for subgroup difference P = 0.48). Studies funded by the manufacturer of either the pMDI or non-pMDI device were available for subgrouping in asthma maintenance (Figure S15 in the Supplementary Information, test for subgroup difference P = 0.22).

## Discussion

In this systematic review of 44 studies, we have found consistent evidence of no clinically meaningful difference between pMDIs and non-pMDI devices (DPIs and SMIs) in the management of asthma and COPD. The certainty of evidence was mainly rated as moderate rather than high due to the presence of some studies with high or unclear risk of bias and the possibility that biases could be operating in the direction of demonstrating equivalence. However, the overwhelming consistency of our findings and the robustness of these results to sensitivity analyses provides a clear conclusion. Results were uniform across a range of different outcomes, not just FEV_1_ and PEFR measurements but also markers of disease control (exacerbations and reliever use) – the latter being important given the greater sensitivity of symptom-based endpoints to detect meaningful differences^31^. Our results are consistent with more focused previous systematic reviews around two decades ago that also showed no differences between devices regarding clinical effectiveness and adverse events^13–16^. Importantly, our study contains updated data from newer studies, new drugs (or drug combinations), as well as a new device type (soft mist inhaler) that were not included in the previous reviews.

Climate change is the greatest global health threat of the twenty-first century^32^, and patients with respiratory diseases are among those projected to be affected disproportionately^33^. Globally, both governments and industry have made commitments to develop more sustainable healthcare systems, with a strong focus on decarbonisation. Increasing the proportion of inhalers without hydrofluorocarbons is an immediate step that can be taken to begin realising these ambitions. Our review supports that such a change may be possible without compromising patient care, though evidently the device class is only one of several important aspects of successful therapeutic changes. We acknowledge that there may be further issues relating to device selection and/or switching at an individual level, which are beyond the scope of this review and cannot be directly answered by our research findings. Furthermore, additional strategies may be employed to reduce inhaler-associated emissions – such as promoting uptake of propellants with a lower carbon footprint, improving inhaler disposal and recycling, reducing reliance on reliever treatments and improving disease control^34–36^.

Selecting the most appropriate inhaler device for an individual patient is a complicated and multifaceted decision. This choice will be influenced by factors including the available agents (e.g. extrafine therapies are only widely available in pMDIs), patient familiarity and preference, patient ability and dexterity, as well as cost and accessibility^37^. Increasingly, clinicians and patients are also being encouraged to consider the environmental impact of inhaler selection in position statements from expert bodies^38^ and decision aids^39^. There are important ethical issues around potentially trading patient preference for population health^40^, and avoiding any shaming of individuals who continue with pMDIs for strong user or disease reasons^41^. Nevertheless, multiple surveys have shown that for most respiratory patients the environmental impact of their inhaler is an important consideration and one that could lead them to seek a device change^42–44^.

When contemplating a widespread move away from pMDIs to other devices (primarily DPIs), two potential concerns should be considered. The first concern is the suitability of DPIs for subgroups with limited lung function (e.g. the very young, the very old, or those experiencing an exacerbation), due to the need to generate sufficient inspiratory flow for adequate drug delivery^45^. However, studies have shown that the majority of patients, including hospitalised patients nearing discharge, can achieve sufficient inspiratory flow to use an appropriately selected DPI^46–48^. A recent systematic review focusing of DPIs for primary school aged children with asthma found that the majority of children could use DPIs with adequate training and support^49^. Nevertheless, there are likely subpopulations for whom a pMDI will be the most appropriate device based on considerations of flow or other technical factors. The second concern is the potential negative impact of changing device type on disease control. Although the CRITIKAL study reported no clear difference between MDIs and DPIs in critical errors rates^50^, interpretation of findings across the wider inhaler-error literature remains challenging due to inconsistent definitions of errors and of what qualifies as ‘critical.’^51^ Any switching of individuals between devices requires investment of time in education and training. Outcomes may be worse if this is not undertaken, but large-scale real-world evidence suggests it can be undertaken successfully^52–55^. Changing inhaler devices does not have a predictable effect on outcomes^56^, and large-scale switching for non-clinical reasons may lead to worsened disease control^57^. Avoiding a loss of disease control is important not only for individual patient benefit, but also for sustainability. The greatest environmental impact of respiratory care comes from poor disease control leading to frequent use of reliever medications, unscheduled healthcare attendance and hospital admissions^36^. A preventer inhaler that an individual can and will use is therefore preferable to one which is “greener” but remains in the cupboard.

Some strengths of our research are the large number of included studies, and the robust methodologies that were employed. Our research does have some limitations. First, to ensure that solely the inhaler device was being compared, we only included studies with equivalent drugs and doses in each arm – this limited the pool of available studies but does increase the confidence in our findings. Second, we acknowledge that participants in clinical trials are not wholly representative of the wider population with asthma or COPD, which potentially casts some doubt on the ability to generalise these findings. However, limiting our assessment to RCTs helped to reduce unmeasured confounders and other influences on results. Additionally, “real world” trials and analyses of primary care databases have also shown non-inferiority of dry powder inhalers in broader populations “treated as” asthma or COPD, giving greater support to our review’s conclusions^52,53,58^. Third, the review contained a number of equivalence or non-inferiority trials, which may potentially lack assay sensitivity and the ability to detect a true difference if one exists^59^. However, the consistency of results across all conditions and subgroups assessed was notable, and the use of meta-analysis increases the combined power of the set of included studies to counter this limitation. Fourth, a number of pharmaceutical companies are presently developing new propellants with lower global warming potential^34^. While these propellants will not alter the validity of our systematic review’s findings, they may reduce the impetus to move patients away from pMDIs for environmental reasons. Finally, for our assessment of acute asthma management we were only able to identify studies focusing on children.

The safest and most productive way to implement greater use of inhalers that do not contain propellants with a high global warming potential is uncertain. This review provides reassurance to patients, clinicians, and researchers that – at least based on RCT data – DPIs and SMIs are equivalent to pMDIs when administering corresponding drugs and doses among subjects trained to satisfactorily use their device. It lays a strong basis upon which to undertake future studies assessing optimal strategies to reduce the overall environmental impact of inhaler therapy.

## Supplementary information

Supplementary Information

## Data Availability

Our research protocol is publicly available on the Monash University research repository (10.26180/26065789.v2). The datasets generated and/or analysed during the current study are also available on the Monash University research repository (10.26180/28916777.v2).
