# Supplementary material for: Efficacy and safety of different inhaler types for asthma and chronic obstructive pulmonary disease. a systematic review and meta-analysis
Source: NPJ Prim Care Respir Med. 2026 Feb 16;36:18. doi: 10.1038/s41533-026-00488-4 (PMC13022311; doi:10.1038/s41533-026-00488-4)
Supplement: Supplementary file 1 — Supplementary Information [file 41533_2026_488_MOESM1_ESM.docx]

**Supplementary Information**

**Loftus et al - Efficacy and Safety of Different Inhaler Types for Asthma and Chronic Obstructive Pulmonary Disease. A Systematic Review and Meta-Analysis.**

Contents

[Section A. Search Strategies 3](#_Toc213418643)

[Table S1 PubMed search strategy 3](#_Toc213418644)

[Table S2 Embase search strategy 4](#_Toc213418645)

[Table S3 Cochrane Central Register of Controlled Trials search strategy 5](#_Toc213418646)

[Section B. Additional methods 6](#_Toc213418647)

[Figure S1 – Risk of Bias assessments for each study 6](#_Toc213418648)

[Table S4 – Summary of Minimal Clinically Important Differences Used for GRADE Assessment 7](#_Toc213418649)

[Table S5 – Funder for each Study 10](#_Toc213418650)

[References 11](#_Toc213418651)

[Section C. Summary of findings 12](#_Toc213418652)

[Table S6 – Summary of findings for asthma maintenance 12](#_Toc213418653)

[Table S7 – Summary of findings for COPD 13](#_Toc213418654)

[Table S8 – Summary of findings for acute asthma exacerbations 14](#_Toc213418655)

[Section D. Additional Results 15](#_Toc213418656)

[FEV_1_ 15](#_Toc213418657)

[Table S9 Asthma maintenance: Additional FEV1 results not included in the meta-analysis 15](#_Toc213418658)

[Figure S2. FEV_1_ in Acute Asthma Exacerbations (% predicted) 15](#_Toc213418659)

[PEFR 15](#_Toc213418660)

[Table S10 Asthma maintenance: Additional PEFR results not included in meta-analysis 15](#_Toc213418661)

[Figure S3. PEFR (L/min) in (A) Acute Asthma Exacerbations and (B) COPD 15](#_Toc213418662)

[Reliever use 16](#_Toc213418663)

[Figure S4 Reliever Use in (A) Asthma Maintenance (SMD) and (B) COPD (puffs/day) 16](#_Toc213418664)

[Table S11 Asthma maintenance: Additional reliever control results not included in meta-analysis 17](#_Toc213418665)

[Symptom control 18](#_Toc213418666)

[Figure S5. Symptom control in (A) Asthma Maintenance (SMD, 8-30 weeks), (B) Acute Asthma Exacerbations (Modified Wood Clinical Asthma Score) and (C) COPD (CAT score) 18](#_Toc213418667)

[Table S12 Asthma maintenance and acute asthma exacerbations: Additional symptom control results not included in meta-analysis for asthma maintenance 19](#_Toc213418668)

[Quality of life 20](#_Toc213418669)

[Figure S6. Quality of life (AQLQ ≥0.5 improved from baseline) in Asthma Maintenance 20](#_Toc213418670)

[Table S13 Asthma maintenance and COPD: Additional quality of life results not included in meta-analysis 20](#_Toc213418671)

[Disease exacerbations 21](#_Toc213418672)

[Figure S7. Disease exacerbations (risk of >1) in (A) Asthma Maintenance and (B) COPD 21](#_Toc213418673)

[Table S14 Asthma maintenance: Additional disease exacerbation results not included in meta-analysis 22](#_Toc213418674)

[Adverse events 23](#_Toc213418675)

[Figure S8. Adverse Events (risk of >1) in (A) Asthma maintenance, (B) Acute Asthma Exacerbations and (C) COPD 23](#_Toc213418676)

[Figure S9. Serious Adverse Events (risk of >1) in (A) Asthma maintenance and (B) COPD 25](#_Toc213418677)

[Figure S10. Treatment-related adverse events (risk of >1) in (A) Asthma maintenance and (B) COPD 26](#_Toc213418678)

[Figure S11. Treatment-related serious adverse events (risk of >1) in (A) Asthma maintenance and (B) COPD 27](#_Toc213418679)

[Mortality 28](#_Toc213418680)

[Figure S12. Mortality in COPD 28](#_Toc213418681)

[Subgroup analyses 29](#_Toc213418682)

[Figure S13. Subgroup analysis of FEV1 by age of participants in Asthma Maintenance 29](#_Toc213418683)

[Figure S14. Subgroup analysis of FEV1 by non-pMDI device type in COPD 30](#_Toc213418684)

[Figure S15. Subgroup analysis of FEV1 by manufacturer funding in Asthma Maintenance 31](#_Toc213418685)

[Section E. Additional studies 32](#_Toc213418686)

[Table S15 – Excluded studies that might appear to meet inclusion criteria 32](#_Toc213418687)

# Section A. Search Strategies

Search strategies were developed by an information specialist (SM). The PubMed search was developed first then translated for Embase and CENTRAL, using appropriate alternative thesaurus terms and search syntax. To test the reliability of the search, we used the studies included in the 2023 systematic review by Montoro et al.^1^ The review by Montoro included 35 studies, 34 of which were included in PubMed. Our PubMed search strategy retrieved all 34 studies. Suggestions for names of common brands of inhalers were sought from clinical members of the review team and supplemented by searches of Google. No search limits were applied regarding date, language of publication or study design, but conference abstracts and trial registry records were excluded.

## Table S1 PubMed search strategy

| **Set** | **Concept** | **Search terms** |
| --- | --- | --- |
| #1 | Asthma or COPD | (Asthma [Mesh] OR Pulmonary Disease, Chronic Obstructive [Mesh] OR asthma[TIAB] OR "chronic obstructive pulmonary disease"[TIAB] OR COPD[TIAB] OR "respiratory disease*"[TIAB]) |
| #2 | Dry-powder inhalers | (Dry Powder Inhalers [Mesh] OR "dry powder inhaler*"[TIAB] OR "dry powdered inhaler*"[TIAB] OR DPI[TIAB] OR DPIs[TIAB] OR "soft mist inhaler*"[TIAB] OR "softmist inhaler*"[TIAB] OR SMI[TIAB] OR SMIs[TIAB] OR Turbuhaler*[TIAB] OR Breezhaler*[TIAB] OR Ellipta[TIAB] OR Diskus[TIAB] OR Genuair[TIAB] OR Accuhaler*[TIAB] OR Handihaler*[TIAB] OR Easyhaler*[TIAB] OR Rotahaler*[TIAB] OR Respimat[TIAB]) |
| #3 | Metered-dose inhalers | (Metered Dose Inhalers [Mesh] OR "metered dose inhaler*"[TIAB] OR "breath-actuated inhaler*"[TIAB] OR "aerosol inhaler*"[TIAB] OR MDI[TIAB] OR MDIs[TIAB] OR pMDI[TIAB] OR pMDIs[TIAB] OR BAI[TIAB] OR BAIs[TIAB]) |
| #4 |  | #1 AND #2 AND #3 |
| #5 | Switching from inhaler type | #1 AND ((#2 AND switch*[TIAB]) OR (#3 AND switch*[TIAB])) |
| #6 |  | #4 OR #5 |

**PubMed search string**

**Search date: 25 September 2025 (1130 records retrieved)**

((Asthma [Mesh] OR Pulmonary Disease, Chronic Obstructive [Mesh] OR asthma[TIAB] OR "chronic obstructive pulmonary disease"[TIAB] OR COPD[TIAB] OR "respiratory disease*"[TIAB]) AND (Dry Powder Inhalers [Mesh] OR "dry powder inhaler*"[TIAB] OR "dry powdered inhaler*"[TIAB] OR DPI[TIAB] OR DPIs[TIAB] OR "soft mist inhaler*"[TIAB] OR "softmist inhaler*"[TIAB] OR SMI[TIAB] OR SMIs[TIAB] OR Turbuhaler*[TIAB] OR Breezhaler*[TIAB] OR Ellipta[TIAB] OR Diskus[TIAB] OR Genuair[TIAB] OR Accuhaler*[TIAB] OR Handihaler*[TIAB] OR Easyhaler*[TIAB] OR Rotahaler*[TIAB] OR Respimat[TIAB]) AND (Metered Dose Inhalers [Mesh] OR "metered dose inhaler*"[TIAB] OR "breath-actuated inhaler*"[TIAB] OR "aerosol inhaler*"[TIAB] OR MDI[TIAB] OR MDIs[TIAB] OR pMDI[TIAB] OR pMDIs[TIAB] OR BAI[TIAB] OR BAIs[TIAB])) OR ((Asthma [Mesh] OR Pulmonary Disease, Chronic Obstructive [Mesh] OR asthma[TIAB] OR "chronic obstructive pulmonary disease"[TIAB] OR COPD[TIAB] OR "respiratory disease*"[TIAB]) AND (((Dry Powder Inhalers [Mesh] OR "dry powder inhaler*"[TIAB] OR "dry powdered inhaler*"[TIAB] OR DPI[TIAB] OR DPIs[TIAB] OR Turbuhaler*[TIAB] OR Breezhaler*[TIAB] OR Ellipta[TIAB] OR Diskus[TIAB] OR Genuair[TIAB] OR Accuhaler*[TIAB] OR Handihaler*[TIAB] OR Easyhaler*[TIAB] OR Rotahaler*[TIAB] OR Respimat[TIAB]) AND switch*[TIAB]) OR ((Metered Dose Inhalers [Mesh] OR "metered dose inhaler*"[TIAB] OR "breath-actuated inhaler*"[TIAB] OR "aerosol inhaler*"[TIAB] OR MDI[TIAB] OR MDIs[TIAB] OR pMDI[TIAB] OR pMDIs[TIAB] OR BAI[TIAB] OR BAIs[TIAB]) AND switch*[TIAB])))

## Table S2 Embase search strategy

**Embase (Ovid) 1947 to September 24, 2025**

| **#** | **Search Statement** | **Results** |
| --- | --- | --- |
| 1 | exp Asthma/ or exp Chronic Obstructive Lung Disease/ or (asthma or chronic obstructive pulmonary disease or COPD or respiratory disease*).ti,ab,kf. | 651047 |
| 2 | Dry Powder Inhaler/ or (dry powder* inhaler* or soft mist inhaler* or softmist inhaler* or SMI or SMIs or DPI or DPIs or turbuhaler* or breezhaler* or ellipta or diskus or genuair or accuhaler* or handihaler* or easyhaler* or rotahaler or respimat).ti,ab,kf. | 35864 |
| 3 | exp Metered Dose Inhalers/ or (metered dose inhaler* or breath-actuated inhaler* or aerosol inhaler* or MDI or MDIs or pMDI or pMDIs or BAI or BAIs).ti,ab,kf. | 26308 |
| 4 | and/1-3 | 3028 |
| 5 | switch*.ti,ab,kf. | 317680 |
| 6 | 1 and ((2 and 5) or (3 and 5)) | 424 |
| 7 | 4 or 6 | 3278 |
| 8 | (note or letter or comment or editorial or review or conference abstract or conference paper or chapter).pt. | 13719253 |
| 9 | 7 not 8 | 1573 |

## Table S3 Cochrane Central Register of Controlled Trials search strategy

**Cochrane Central Register of Controlled Trials (Cochrane Library) Issue 8 of 12, August 2025**

| **#** | **Search** | **Hits** |
| --- | --- | --- |
| 1 | MeSH descriptor: [Asthma] explode all trees | 14730 |
| 2 | MeSH descriptor: [Pulmonary Disease, Chronic Obstructive] explode all trees | 8159 |
| 3 | (asthma or "chronic obstructive pulmonary disease" or COPD or "respiratory disease*"):ti,ab,kw (Word variations have been searched) | 60391 |
| 4 | #1 or #2 or #3 | 61165 |
| 5 | MeSH descriptor: [Dry Powder Inhalers] explode all trees | 204 |
| 6 | ((dry NEXT powder* NEXT inhaler*) or DPI or DPIs or (soft NEXT mist NEXT inhaler*) or (softmist NEXT inhaler*) or SMI or SMIs or turbuhaler* or breezhaler* or ellipta or diskus or genuair or accuhaler* or handihaler* or easyhaler* or rotahaler or respimat):ti,ab,kw (Word variations have been searched) | 5837 |
| 7 | #5 or #6 | 5837 |
| 8 | MeSH descriptor: [Metered Dose Inhalers] explode all trees | 537 |
| 9 | ((metered NEXT dose NEXT inhaler*) or (breath NEXT actuated NEXT inhaler*) or (aerosol NEXT inhaler*) or MDI or MDIs or pMDI or pMDIs or BAI or BAIs):ti,ab,kw (Word variations have been searched) | 6184 |
| 10 | #8 or #9 | 6194 |
| 11 | #4 and # 7 and #10 | 988 |
| 12 | (switch*):ti,ab,kw (Word variations have been searched) | 21893 |
| 13 | ((#7 and #12) or (#10 and #12)) | 130 |
| 14 | #11 or #13 | 1089 |
| 15 | ("conference proceeding" or "trial registry record"):pt | 852975 |
| 16 | #14 not #15 in Trials | 584 |

# Section B. Additional methods

## Figure S1 – Risk of Bias assessments for each study


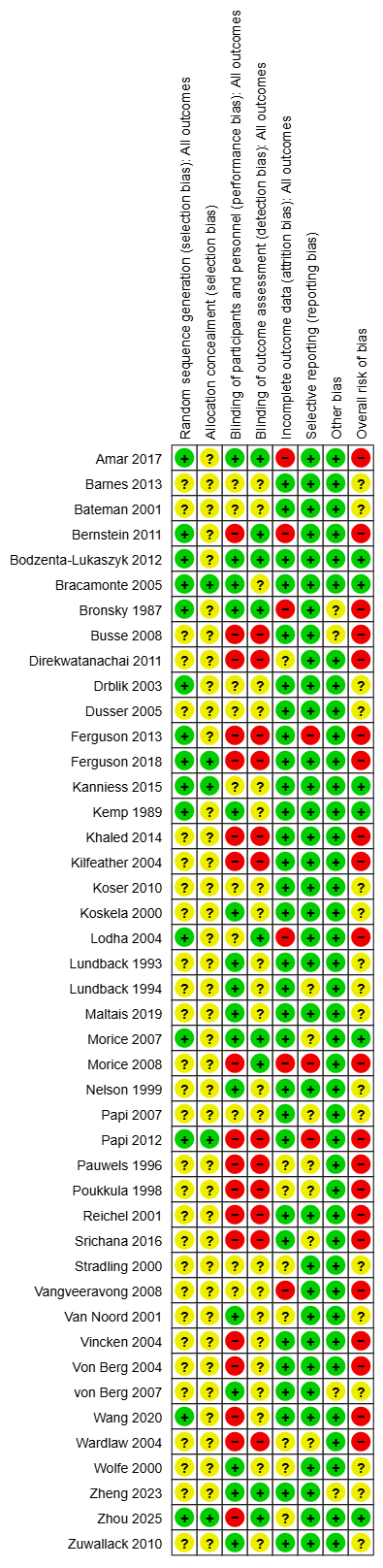

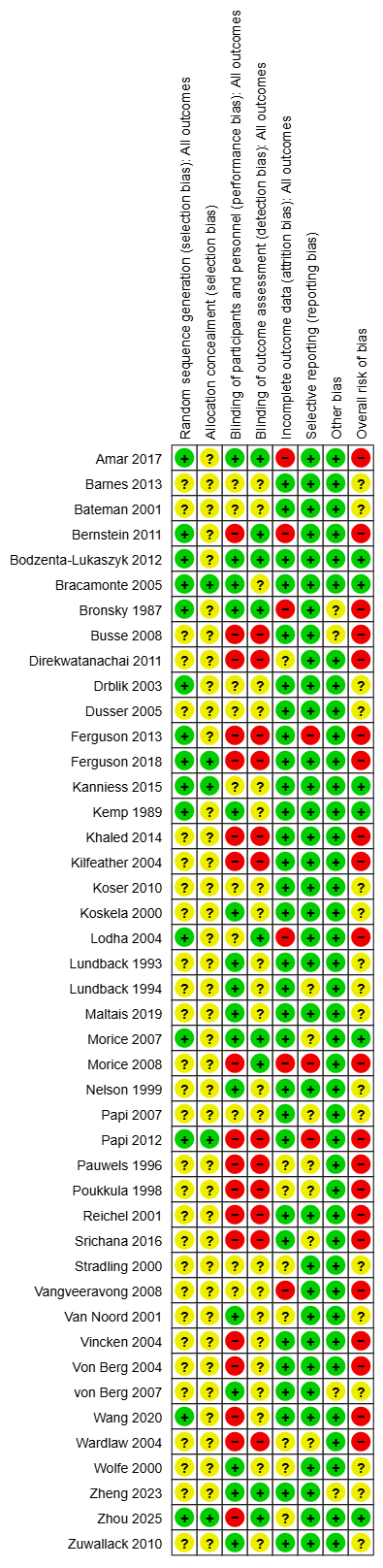

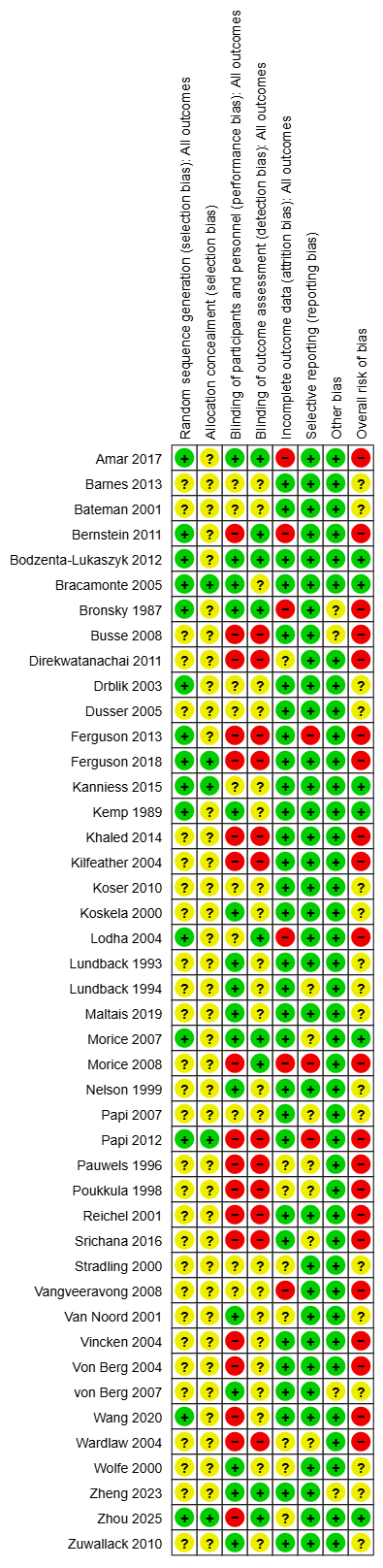


## Table S4 – Summary of Minimal Clinically Important Differences Used for GRADE Assessment

#### Asthma – Maintenance

| **Parameter** | **MCID used for GRADE assessment** | **Source** | **Comments** |
| --- | --- | --- | --- |
| FEV_1_ | 12% predicted value | Bonini et al, 2020 ^2^  Pellegrino et al, 2005 ^3^ | Expert consensus report (Bonini et al, 2020) suggests ≥15% for long-term trials (years) and ≥20% for short-term trials (weeks).  Of note these are consensus values without strong original evidence to support them.  We have opted for the more conservative 12% value cited in Pellegrino et al, 2005. |
| Peak Expiratory Flow Rate (PEFR) | 18.8L/min | Santanello et al, 1999 ^4^ |  |
| Asthma Control Questionnaire (ACQ) | 0.5 points | Bonini et al, 2020 ^2^ | Consensus value |
| Reliever use | 0.81 puffs/day | Santanello et al, 1999 ^4^ |  |
| Exacerbations | 5% (absolute risk difference) | Bonini et al, 2020 ^2^ | Consensus value. A reduction in *annual* exacerbation rate or in the risk of having a severe asthma-related event ranging from 20–40% for a given asthma treatment regimen and/or intervention is considered clinically relevant in RCTs.  Given median follow up of 12 weeks for included studies, we have taken the conservative estimate of 5% (will make it less likely for no difference to be shown) |
| Adverse events | 5% (absolute risk difference) | Expert opinion | No published MCID estimates/values – determined following discussion with respiratory experts on authorship team, to permit GRADE assessment |

#### Asthma – Acute

| **Parameter** | **MCID used for GRADE assessment** | **Source** | **Comments** |
| --- | --- | --- | --- |
| FEV_1_ | 12% predicted value | Extrapolated from asthma maintenance ( Pellegrino et al, 2005)^3^ | See comments above under Asthma maintenance. |
| Peak Expiratory Flow Rate (PEFR) | 12% predicted value | Karras et al, 2000 ^5^ | The two included acute asthma studies reporting PEFR did so using *absolute* PEFR values (not % predicted values). Both were paediatric studies.  We have conservatively estimated that the end of treatment PEFRs (approx. 208L/min in both arms) were those individuals’ baseline values (a conservative assumption, as values immediately post-exacerbation may be lower than baseline). Using the Karras et al percent predicted MCID; 12% of 208L/min is 24.96, rounded up to 25L/min.  NB - this estimate would not apply to other groups (e.g. adults) |
|  | 25L/min | Expert opinion (and only relevant to the studies included in this review) |  |
| Modified Wood Clinical Asthma Score | 1 point | Expert opinion  (Influenced by Duarte-Dorado et al, 2013) ^6^ | No published MCID estimates/values. Duarte-Dorado showed 2-point difference between patients admitted to PICU versus regular ward. Inter-rater agreement showed that difference of 0.5 between assessors was common.  Hence in discussion with respiratory experts on authorship team have determined an MCID estimate of 1 point. |
| Adverse events | 5% (absolute risk difference) | Expert opinion | See comments above under Asthma maintenance. |

#### COPD

| **Parameter** | **MCID used for GRADE assessment** | **Source** | **Comments** |
| --- | --- | --- | --- |
| FEV_1_ | 100 mL | Jones et al, 2014 ^7^  Cazzola et al, 2008 ^8^ |  |
| PEFR | 18.8L/min | Expert opinion | No published MCID estimates/values. Have extrapolated from asthma maintenance to permit GRADE assessment. |
| Exacerbations | 20% (absolute change) | Expert opinion (influenced by Jones et al, 2014) ^7^ | Jones et al states no validated MCID. Have used widely suggested value of 20% to permit GRADE assessment. |
| CAT score (COPD assessment test) | 2 points | Kon et al, 2014 ^9^ |  |
| Reliever use | 0.81puffs/day | Expert opinion | No published MCID estimates/values. Have extrapolated from asthma maintenance to permit GRADE assessment. |
| Adverse events | 5% (absolute risk difference) | Expert opinion | See comments above under Asthma maintenance. |
| Mortality | 1% (absolute risk difference) | Expert opinion | No published MCID estimates/values – decided after discussion with respiratory experts on authorship team, to permit GRADE assessment. |

## Table S5 – Funder for each Study

| **Publication** | **Manufacturer funded?** | **Company** | **Which arm(s)?** |
| --- | --- | --- | --- |
| Asthma Maintenance | | | |
| Amar 2017 | Yes | Merck & Co. | DPI only |
| Barnes 2013 | Yes | Chiesi | pMDI only |
| Bateman 2001 | Yes | Glaxo Wellcome | DPI only |
| Bernstein 2011 | Yes | Merck & Co. | pMDI only |
| Bodzenta-Lukaszyk 2012 | Yes | Mundipharma Research Limited | pMDI only |
| Bracamonte 2005 | Yes | GlaxoSmithKline | DPI only |
| Bronsky 1987 | Yes | Glaxo | DPI only |
| Busse 2008 / O'Connor 2010 | Yes | AstraZeneca | pMDI only |
| Dusser 2005 | Yes | Chiesi | pMDI only |
| Kanniess 2015 | Yes | Chiesi | Both |
| Kemp 1989 | Unclear* | Unclear* | Unclear* |
| Koskela 2000 | Yes | Orion Pharma | DPI only |
| Lundback 1993 | Yes | Glaxo | DPI only |
| Lundback 1994 | Yes | Glaxo | DPI only |
| Morice 2007 | Yes | AstraZeneca | Both |
| Morice 2008 | Yes | AstraZeneca | Both |
| Nelson 1999 | Yes | Dura Pharmaceuticals | DPI only |
| Papi 2007 | Yes | Chiesi | pMDI only |
| Papi 2012 | Yes | Chiesi | pMDI only |
| Pauwels 1996 | Yes | Astra Draco | DPI only |
| Poukkula 1998 | Yes | Orion Pharma | DPI only |
| Reichel 2001 | Yes | 3M pharmaceuticals | pMDI only |
| Srichana 2016 | No | N/A | N/A |
| Stradling, 2000 | Yes | Innovata Biomed Ltd | DPI only |
| Van Noord 2001 | Yes | Glaxo | DPI only |
| Vincken 2004 | Yes | Boehringer Ingelheim | SMI only |
| von Berg 2004 | Yes | Boehringer Ingelheim | SMI only |
| Von Berg 2007 | Yes | ATLANTA Pharma | pMDI only |
| Wardlaw 2004 | Yes | Schering-Plough | DPI only |
| Wolfe 2000 | Yes | Glaxo | DPI only |
| Zheng 2023 | Yes | Chiesi | DPI only |
| Zhoud 2025 | No | N/A | N/A |
|  | | | |
| Asthma Acute | | | |
| Direkwatanachai 2011 | Yes | Harn Thai | DPI only |
| Drblik 2003 | Yes | AstraZeneca | DPI only |
| Khaled 2014 | No | N/A | N/A |
| Lodha 2004 | No | N/A | N/A |
| Vangveeravong 2008 | No | N/A | N/A |
|  | | | |
| COPD | | | |
| Ferguson 2013 | Yes | Boehringer Ingelheim | SMI only |
| Ferguson 2018 | Yes | Pearl (member of AstraZeneca Group) | DPI only |
| Kilfeather 2004 | Yes | Boehringer Ingelheim | SMI only |
| Koser 2010 | Yes | GlaxoSmithKline | DPI only |
| Maltais 2019 | Yes | AstraZeneca | MDI only |
| Wang 2020 | Yes | AstraZeneca | Both |
| Zuwallack 2010 | Yes | Boehringer Ingelheim | Both |

* No information available concerning funding for this study. A Glaxo device (Rotahaler) was used in the DPI arm.

## References

1 Montoro J, Antolín-Amérigo D, Izquierdo-Domínguez A, Zapata JJ, González G, Valero A. Impact of Asthma Inhalers on Global Climate: A Systematic Review of Their Carbon Footprint and Clinical Outcomes in Spain. *J Investig Allergol Clin Immunol*. 2023; **33**: 250-62.

2 Bonini M, Di Paolo M, Bagnasco D, Baiardini I, Braido F, Caminati M*, et al.* Minimal clinically important difference for asthma endpoints: an expert consensus report. *Eur Respir Rev*. 2020; **29**.

3 Pellegrino R, Viegi G, Brusasco V, Crapo RO, Burgos F, Casaburi R*, et al.* Interpretative strategies for lung function tests. *Eur Respir J*. 2005; **26**: 948-68.

4 Santanello NC, Zhang J, Seidenberg B, Reiss TF, Barber BL. What are minimal important changes for asthma measures in a clinical trial? *Eur Respir J*. 1999; **14**: 23-7.

5 Karras DJ, Sammon ME, Terregino CA, Lopez BL, Griswold SK, Arnold GK. Clinically meaningful changes in quantitative measures of asthma severity. *Acad Emerg Med*. 2000; **7**: 327-34.

6 Duarte-Dorado DM, Madero-Orostegui DS, Rodriguez-Martinez CE, Nino G. Validation of a scale to assess the severity of bronchiolitis in a population of hospitalized infants. *J Asthma*. 2013; **50**: 1056-61.

7 Jones PW, Beeh KM, Chapman KR, Decramer M, Mahler DA, Wedzicha JA. Minimal clinically important differences in pharmacological trials. *Am J Respir Crit Care Med*. 2014; **189**: 250-5.

8 Cazzola M, MacNee W, Martinez FJ, Rabe KF, Franciosi LG, Barnes PJ*, et al.* Outcomes for COPD pharmacological trials: from lung function to biomarkers. *Eur Respir J*. 2008; **31**: 416-69.

9 Kon SS, Canavan JL, Jones SE, Nolan CM, Clark AL, Dickson MJ*, et al.* Minimum clinically important difference for the COPD Assessment Test: a prospective analysis. *Lancet Respir Med*. 2014; **2**: 195-203.

# Section C. Summary of findings

## Table S6 – Summary of findings for asthma maintenance

| Outcome and follow-up | Patients (studies), N | Relative effect (95% CI) | **Absolute effects (95% CI)** | | | Certainty | What happens |
| --- | --- | --- | --- | --- | --- | --- | --- |
|  |  |  | **pMDIs** | **non-pMDIs** | **Difference** |  |  |
| FEV_1_, % predicted Follow-up: range 4 weeks to 52 weeks | 9958 (29 RCTs) | - | 83.18 % | **83.89 %** | **0.71** (0 to 1.42) | ⨁⨁⨁◯ Moderate^a^ | Non-pMDIs probably result in little to no difference in FEV_1_. |
| PEFR, L/min Follow-up: range 4 weeks to 30 weeks | 8860 (26 RCTs) | - | 400.15 L/min | **401.22 L/min** | **1.07** (-0.93 to 3.06) | ⨁⨁⨁◯ Moderate^a^ | Non-pMDIs probably result in little to no difference in PEFR. |
| Symptom control, ACQ-7 Follow-up: range 8 weeks to 30 weeks | 3836 (8 RCTs) | - | 0 points (change from baseline) | **-0.024 points (change from baseline)** | **-0.024** (-0.05 to 0.01) | ⨁⨁⨁◯ Moderate^a^ | Non-pMDIs may result in little to no difference in symptom control. |
| Quality of life, AQLQ ≥0.5 improved from baseline Follow-up: range 12 weeks to 30 weeks | 871 (2 RCTs) | **RR = 1.02** (0.91 to 1.14) | 575 per 1,000 | **586 per 1,000** (523 to 655) | **11 more per 1,000** (from 52 fewer to 80 more) | ⨁◯◯◯ Very low^a,b^ | The evidence is very uncertain about the effect of non-pMDIs on quality of life. |
| Reliever use, puffs/day Follow-up: range 4 weeks to 30 weeks | 4308 (13 RCTs) | - | 1.27 puffs/day | **1.32 puffs/day** | **0.05** (-0.16 to 0.23) | ⨁⨁⨁◯ Moderate^a^ | Non-pMDIs probably result in little to no difference in reliever use. |
| Exacerbations, No. with at least one Follow-up: range 4 weeks to 52 weeks | 7532 (19 RCTs) | **RR = 0.87** (0.72 to 1.05) | 59 per 1,000 | **52 per 1,000** (43 to 62) | **8 fewer per 1,000** (from 17 fewer to 3 more) | ⨁⨁⨁◯ Moderate^a^ | Non-pMDIs probably result in little to no difference in exacerbations. |
| Mortality Follow-up: range 4 to 52 weeks | 4700 (12 RCTs) | No deaths were reported. | | | | - |  |
| Adverse events, No. with at least one Follow-up: range 4 weeks to 52 weeks | 8519 (25 RCTs) | **RR = 0.98** (0.94 to 1.02) | 359 per 1,000 | **352 per 1,000** (338 to 366) | **7 fewer per 1,000** (from 22 fewer to 7 more) | ⨁⨁⨁◯ Moderate^a^ | Non-pMDIs probably result in little to no difference in adverse events. |
| **CI:** confidence interval; **MD:** mean difference; **RR:** risk ratio  a. Most included studies at high or unclear risk of bias.  b. Confidence intervals include the possibility of both a meaningful benefit and a meaningful harm | | | | | | | |

## Table S7 – Summary of findings for COPD

| Outcome and follow-up | Patients (studies), N | Relative effect (95% CI) | **Absolute effects (95% CI)** | | | Certainty | What happens |
| --- | --- | --- | --- | --- | --- | --- | --- |
|  |  |  | **pMDIs** | **non-pMDIs** | **Difference** |  |  |
| FEV_1_, L Follow-up: range 12 weeks to 48 weeks | 3946 (7 RCTs) | - | 0.23 L (change from baseline) | **0.24 L (change from baseline)** | **0.01** (-0.01 to 0.02) | ⨁⨁⨁◯ Moderate^a^ | Non-pMDIs probably result in little to no difference in FEV_1_. |
| PEFR, L/min Follow-up: 12 weeks | 644 (2 RCTs) | - | 232.35 L/min | **229.79 L/min** | **-2.56** (-9.17 to 4.05) | ⨁⨁⨁◯ Moderate^a^ | Non-pMDIs probably result in little to no difference in PEFR. |
| Symptom control, CAT score Follow-up: 24 weeks | 1006 (1 RCT) | - | -3.56 points (change from baseline) | **-4.15 points (change from baseline)** | **-0.59** (-1.19 to 0.01) | ⨁⨁⨁◯ Moderate^a^ | Non-pMDIs probably result in little to no difference in symptom control. |
| Quality of life | 871 (2 RCTs) | Two studies reported this outcome but the results could not be pooled. | | | | ⨁◯◯◯ Very low^a,b,c^ | The evidence is very uncertain about the effect of non-pMDIs on quality of life. |
| Reliever use, puffs/day Follow-up: range 12 weeks to 24 weeks | 1265 (3 RCTs) | - | 2.3 puffs/day | **2.09 puffs/day** | **-0.21** (-0.51 to 0.1) | ⨁⨁⨁◯ Moderate^a^ | Non-pMDIs probably result in little to no difference in reliever use. |
| Exacerbations, No. with at least one Follow-up: range 12 weeks to 48 weeks | 4101 (7 RCTs) | **RR = 1.08** (0.94 to 1.24) | 147 per 1,000 | **159 per 1,000** (139 to 183) | **12 more per 1,000** (from 9 fewer to 35 more) | ⨁⨁⨁◯ Moderate^a^ | Non-pMDIs probably result in little to no difference in exacerbations. |
| Mortality Follow-up: range 12 weeks to 48 weeks | 3657 (6 RCTs) | **RR = 1.40** (0.59 to 3.32) | 5 per 1,000 | **8 per 1,000** (3 to 18) | **2 more per 1,000** (from 2 fewer to 12 more) | ⨁⨁◯◯ Low^a,c^ | Non-pMDIs may result in little to no difference in mortality. |
| Adverse events, No. with at least one Follow-up: range 12 weeks to 48 weeks | 4106 (7 RCTs) | **RR = 1.02** (0.94 to 1.11) | 496 per 1,000 | **506 per 1,000** (466 to 551) | **10 more per 1,000** (from 30 fewer to 55 more) | ⨁⨁◯◯ Low^a,c^ | Non-pMDIs may result in little to no difference in adverse events. |
| **CI:** confidence interval; **MD:** mean difference; **RR:** risk ratio  a. Included studies at high or unclear risk of bias.  b. Studies were not pooled, therefore precision and heterogeneity could not be estimated. The studies differed in their direction of effect.  c. Confidence intervals include the possibility of both little or no effect and a meaningful harm. | | | | | | | |

## Table S8 – Summary of findings for acute asthma exacerbations

| Outcome and follow-up | Patients (studies), N | Relative effect (95% CI) | **Absolute effects (95% CI)** | | | Certainty | What happens |
| --- | --- | --- | --- | --- | --- | --- | --- |
|  |  |  | **pMDIs** | **non-pMDIs** | **Difference** |  |  |
| FEV_1_, % predicted Follow-up: 60 minutes | 103 (1 RCT) | - | 66.7 % | **68.7 %** | **2** (-2.9 to 6.9) | ⨁⨁◯◯ Low^a,b^ | Non-pMDIs may result in little to no difference in FEV_1_. |
| PEFR, L/min Follow-up: 30 minutes | 259 (2 RCTs) | - | 207.5 L/min | **208.88 L/min** | **1.38** (-17.97 to 20.73) | ⨁⨁◯◯ Low^a,b^ | Non-pMDIs may result in little to no difference in PEFR. |
| Symptom control, Modified Wood Clinical Asthma Score Follow-up: 60 minutes | 36 (1 RCT) | - | 1.6 points | **1.5 points** | **-0.1** (-0.72 to 0.52) | ⨁⨁◯◯ Low^a,b^ | Non-pMDIs may result in little to no difference in symptom control. |
| Symptom control | (0 studies) | No studies reported symptom control. | | | |  |  |
| Quality of life | (0 studies) | No studies reported quality of life. | | | |  |  |
| Reliever use | (0 studies) | No studies reported reliever use. | | | |  |  |
| Mortality | (0 studies) | No deaths were reported | | | |  |  |
| Adverse events, No. with at least one | 36 (1 RCT) | **RR = 0.33** (0.04 to 2.91) | 56 per 1,000 | **18 per 1,000** (2 to 162) | **37 fewer per 1,000** (from 53 fewer to 106 more) | ⨁◯◯◯ Very low^a,b,c^ | The evidence is very uncertain about the effect of non-pMDIs on adverse events. |
| **CI:** confidence interval; **MD:** mean difference; **RR:** risk ratio  a. Included studies at high or unclear risk of bias.  b. Insufficient information for precision, based on too few participants.  c. Confidence interval includes the possibility of both important benefit and important harm. | | | | | | | |

# Section D. Additional Results

## FEV_1_

### Table S9 Asthma maintenance: Additional FEV1 results not included in the meta-analysis

| **Study ID** | **Timepoint** | **Outcome measure** | **Effect estimate** | **Direction** |
| --- | --- | --- | --- | --- |
| Kemp 1989 | Visit 7 (<12 weeks) | Change in FEV_1_ (% predicted) between Hour 0 and Hour 8 | Non-pMDI mean: 7.89  pMDI mean: 6.85  No measure of variance reported. | Better with non-pMDI. |

### Figure S2. FEV_1_ in Acute Asthma Exacerbations (% predicted)


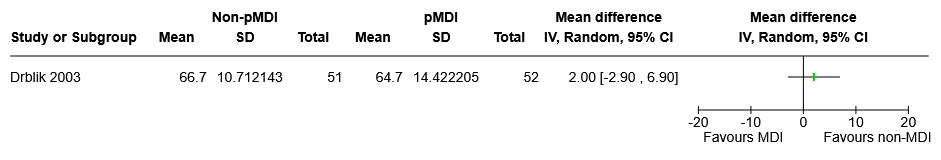


## PEFR

### Table S10 Asthma maintenance: Additional PEFR results not included in meta-analysis

| **Study ID** | **Timepoint** | **Outcome measure** | **Effect estimate** | **Direction of effect** |
| --- | --- | --- | --- | --- |
| Pauwels 1996 | 52 weeks | Risk of at least one ‘event’ (2 consecutive days PEF <80% baseline) | RR 0.80  (95% CI 0.68 to 0.95) | Better with non-pMDI. |

### Figure S3. PEFR (L/min) in (A) Acute Asthma Exacerbations and (B) COPD

**A**


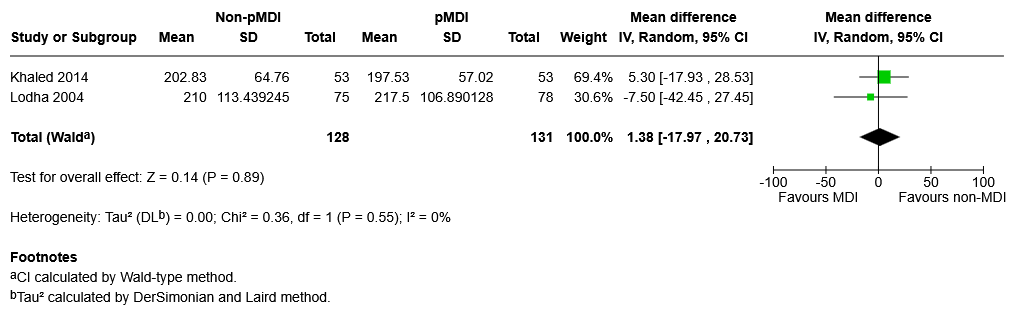


**B**


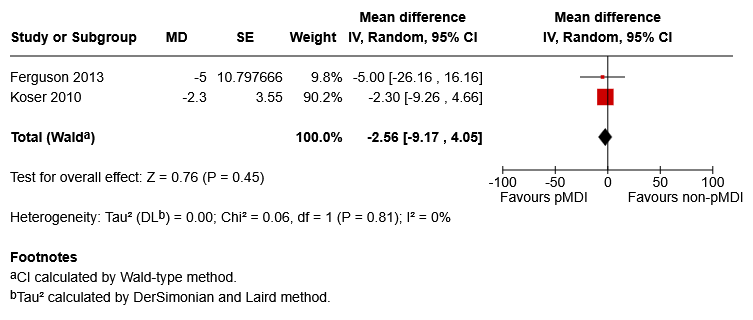


N = 644

## Reliever use

### Figure S4 Reliever Use in (A) Asthma Maintenance (SMD) and (B) COPD (puffs/day)

**A**


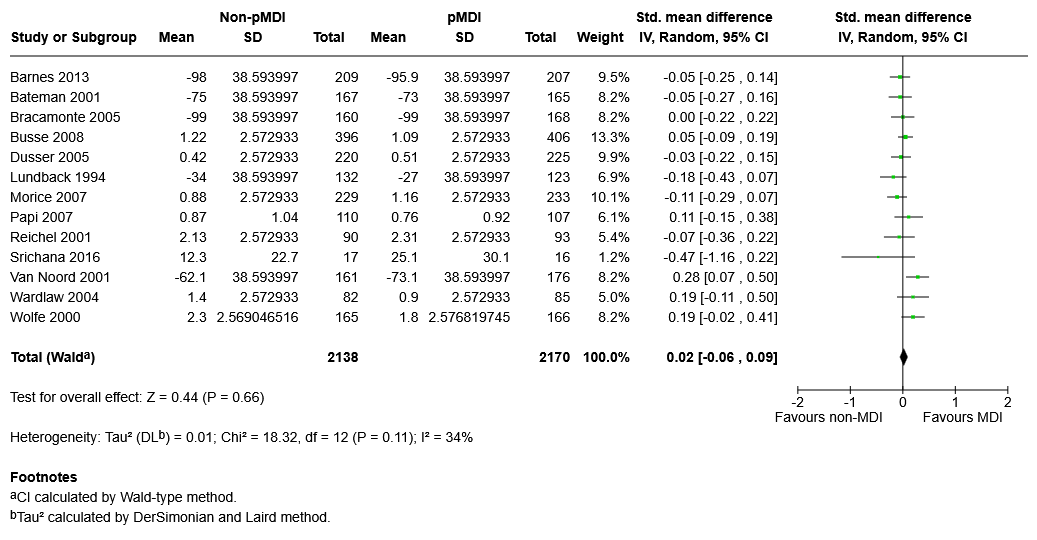


**B**


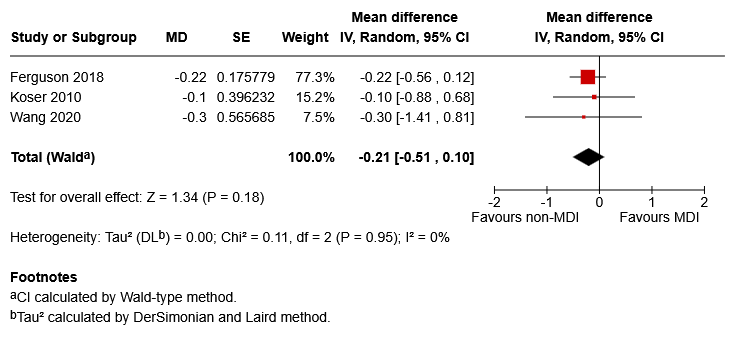


N = 1265

### Table S11 Asthma maintenance: Additional reliever control results not included in meta-analysis

| **Study ID** | **Timepoint** | **Outcome measure** | **Effect estimate** | **Direction of effect** |
| --- | --- | --- | --- | --- |
| Bernstein 2011 | 12 weeks | Change in proportion of nights with nocturnal awakenings due to asthma that required use of a SABA | MD -0.02 (no measure of variance) | Better with non-pMDIs |
| Bodzenta-Lukaszyk 2012 | 12 weeks | Change in rescue medication free days | Least square MD  -2.58% (95% CI -10.25% to 5.09%) | Worse with non-pMDIs |
| Kanniess 2015 | 12 weeks | Change in average use of rescue medication (number of inhalations/day) | MD 0 (95%CI -0.09 to 0.08) | No difference |
| Lundback 1993 | 6 weeks | Patients with same/reduced requirement for rescue medication-days (higher is better) | RR 1.0 (95%CI 0.91 to 1.11) | No difference |
| Papi 2012 | 24 weeks | Day time: Number of patients with inhaled rescue salbutamol-free days in study period (6/12) (higher is better) | RR 0.94 (95%CI 0.77 to 1.16) | Worse with non-pMDIs |
| Von Berg 2007 | 12 weeks | Change in reliever use (% puffs per day) | MD -0.06% (no measure of variance) | Better with pMDI |
| Zheng 2023 | 12 weeks | Change in rescue medication-free days | MD -0.29 (95%CI -1.27 to 0.7) | Worse with non-pMDIs |

## Symptom control

### Figure S5. Symptom control in (A) Asthma Maintenance (SMD, 8-30 weeks), (B) Acute Asthma Exacerbations (Modified Wood Clinical Asthma Score) and (C) COPD (CAT score)

**A**


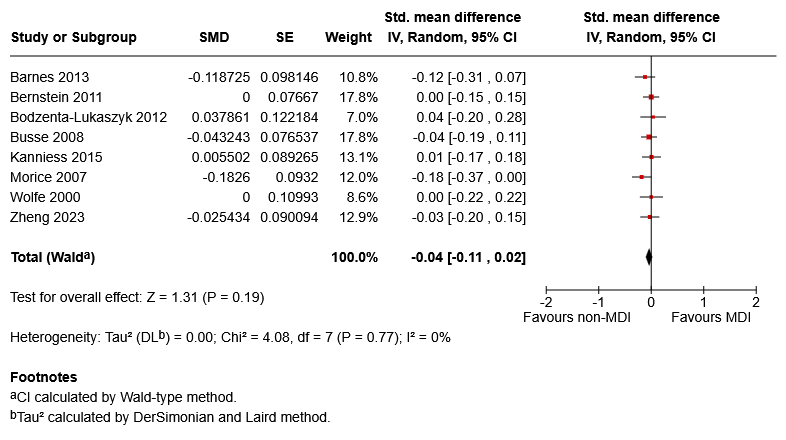


N = 3836

**B**


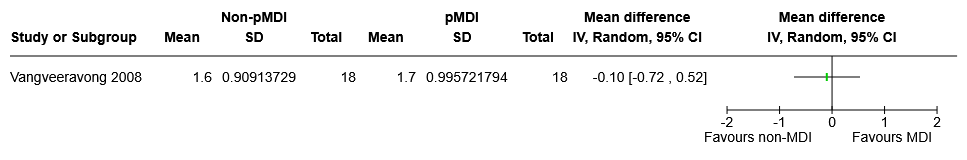


**C**


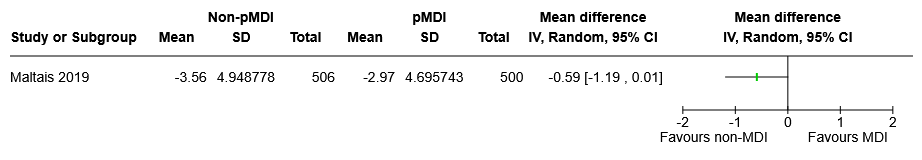


### Table S12 Asthma maintenance and acute asthma exacerbations: Additional symptom control results not included in meta-analysis for asthma maintenance

| **Study ID** | **Timepoint** | **Outcome measure** | **Effect estimate** | **Direction of effect** |
| --- | --- | --- | --- | --- |
| **Asthma maintenance** | | | | |
| Bateman 2001 | 12 weeks | People with symptom free days | RR 0.94 (95% CI 0.77 to 1.15) | Worse with non-pMDI |
| Lundback 1994 | 4 weeks | People with median day symptom score >2 (lower is better) | RR 0.75 (95% CI 0.38 to 1.49) | Better with non-pMDI |
| Papi 2012 | 26 weeks | Controlled & partly controlled asthma (defined using a composite) (%) (higher is better) | RR 1.01 (95% CI, 0.94 to 1.10) | Better with non-pMDI |
| Poukkula 1998 | 12 weeks | Severity sum scores (lower is better) | MD 0.9 (no measure of variance) | Worse with non-pMDI |
| Srichana 2016 | 13 weeks | Symptom free days during study period | MD 6.6 (95% CI ‑10.64 to 23.84) | Better with non-pMDI |
| Van Noord 2001 | 12 weeks | Symptom-free days (higher is better) | Difference in medians -12 (no measure of variance) | Worse with non-pMDI |
| Von Berg 2007 | 12 weeks | Asthma symptom score sum (0-5 scale, lower is better) | MD 0 (assumed reported figures are means, no measure of variance) | No difference |
| Zhou 2025 | 4 weeks | Asthma Control | MD -0.37 (95% CI ‑1.12 to 0.38] | Words with non-pMDI |
| **Acute asthma exacerbations** | | | | |
| Direkwatanachai 2011 | 60 min | Modified Wood Clinical Asthma Score – no. with a score that has fallen by >=50%; or raw score <=3 | Relative risk 1.00 [0.81, 1.23] | None |

## Quality of life

### Figure S6. Quality of life (AQLQ ≥0.5 improved from baseline) in Asthma Maintenance


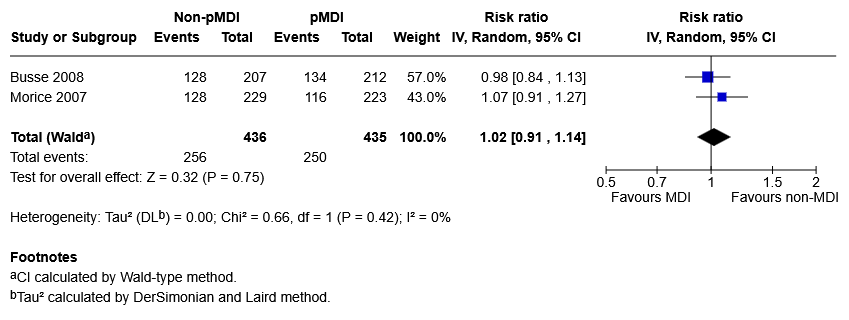


### Table S13 Asthma maintenance and COPD: Additional quality of life results not included in meta-analysis

| **Study ID** | **Timepoint** | **Outcome measure** | **Effect estimate** | **Direction of effect** |
| --- | --- | --- | --- | --- |
| **Asthma maintenance** | | | | |
| Amar 2017 | 12 weeks | PAQLQ[S] | Mean in pMDI group 0.35 (range 0.23 to 0.48). No data reported for non-pMDI group. | Unknown |
| Bernstein 2011 | 12 weeks | AQLQ[S] | MD 0 (no measure of variance) (difference in least square means) | No difference |
| Bodzenta-Lukaszyk 2012 | 12 weeks | Change in AQLQ[S] | MD 0  (95% CI -0.2 to 0.1) | No difference |
| Koskela 2000 | 8 weeks | SGRQ (Parts I–II) | Difference in medians: -0.8 (no measure of variance) | Better with non-pMDI |
| Von Berg 2007 | 12 weeks | PAQLQ(S) | MC 0.19 (no measure of variance) | Better with non-pMDI |
| **COPD** | | | | |
| Ferguson 2018 | 24 weeks | SGRQ (proportion achieving MID ≥4 units) | RD -2 (95% CI -10.18 to 6.19) | Better with non-pMDI |
| Wang 2020 | 12-24 weeks | SGRQ | MD 1 (95% CI -3.14 to 5.14) (difference in least square means) | Worse with non-pMDI |

## Disease exacerbations

### Figure S7. Disease exacerbations (risk of >1) in (A) Asthma Maintenance and (B) COPD

**A**


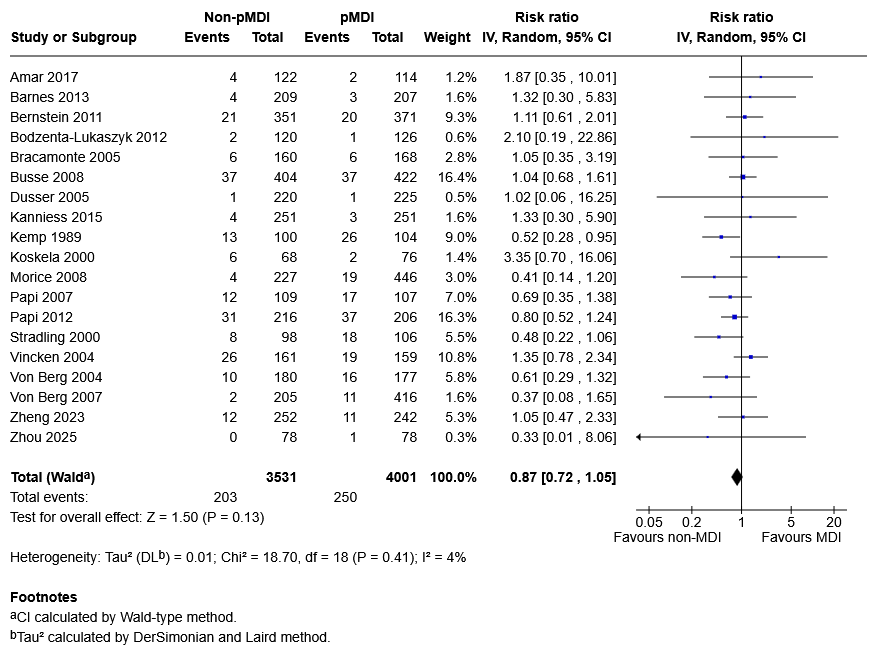


**B**


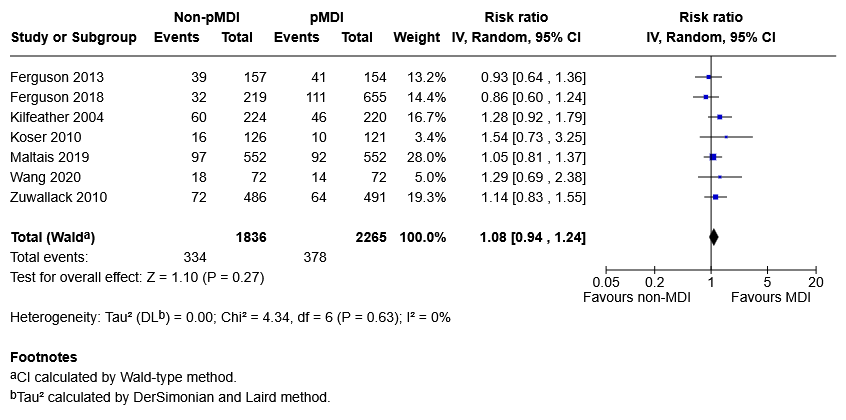


### Table S14 Asthma maintenance: Additional disease exacerbation results not included in meta-analysis

| **Study ID** | **Timepoint** | **Outcome measure** | **Effect estimate** | **Direction of effect** |
| --- | --- | --- | --- | --- |
| Bateman 2001 | 12 weeks | Asthma resulting in emergency treatment, hospitalization, or treatment with additional (excluded) asthma medication (eg, systemic glucocorticoids) | Both groups: 2-3% | Unknown |

## Adverse events

### Figure S8. Adverse Events (risk of >1) in (A) Asthma maintenance, (B) Acute Asthma Exacerbations and (C) COPD

**A**


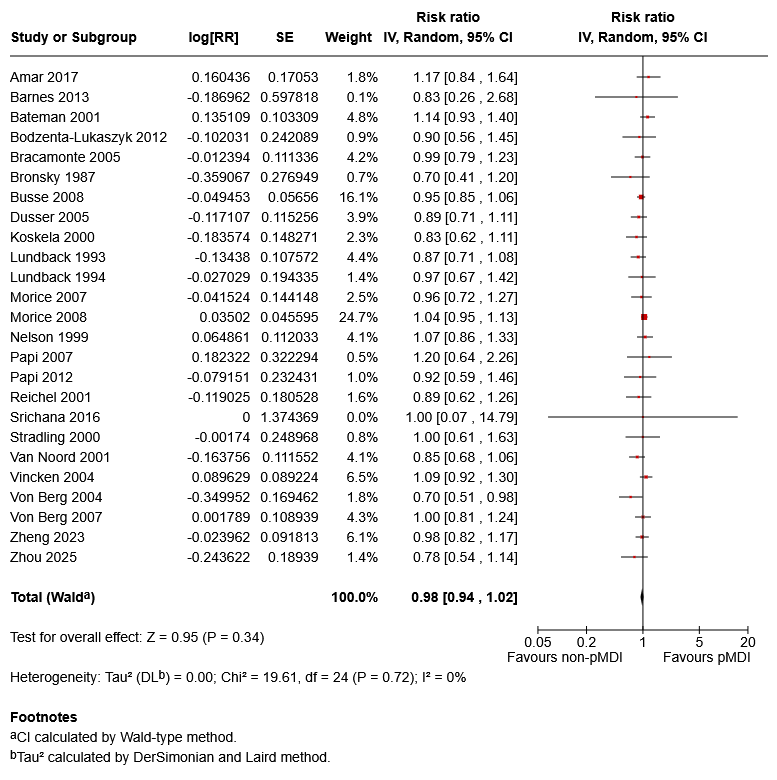
**B**

N = 8723


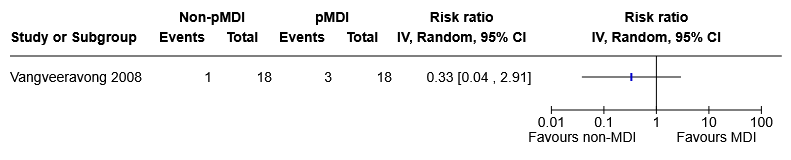


**C**


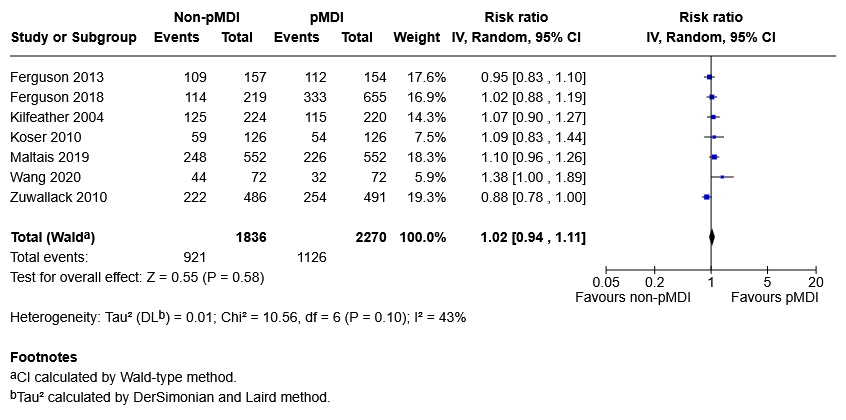


### Figure S9. Serious Adverse Events (risk of >1) in (A) Asthma maintenance and (B) COPD

**A**


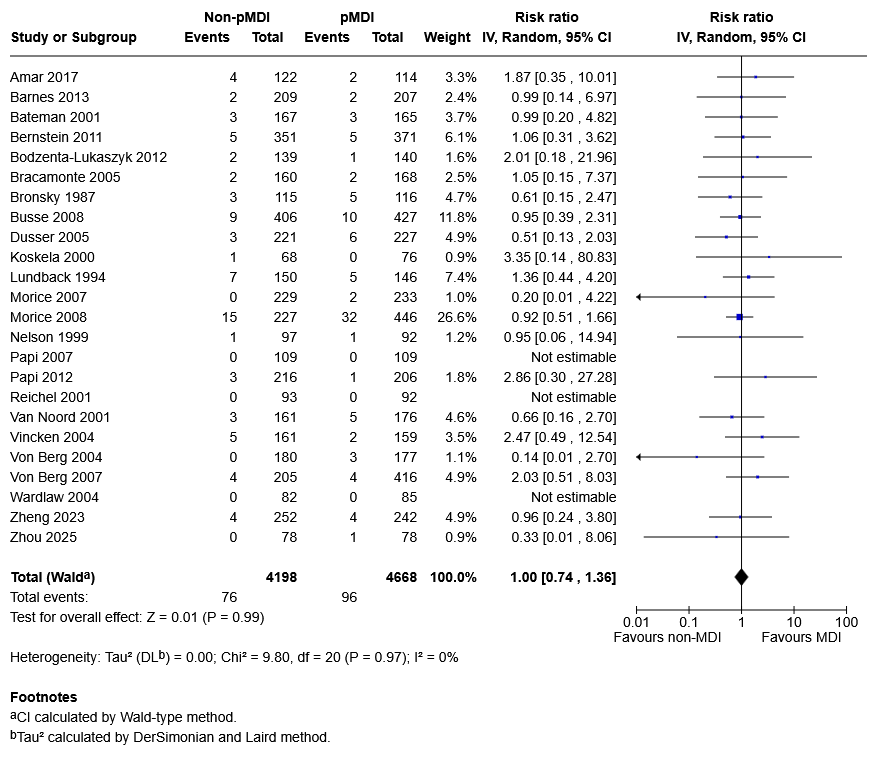


**B**


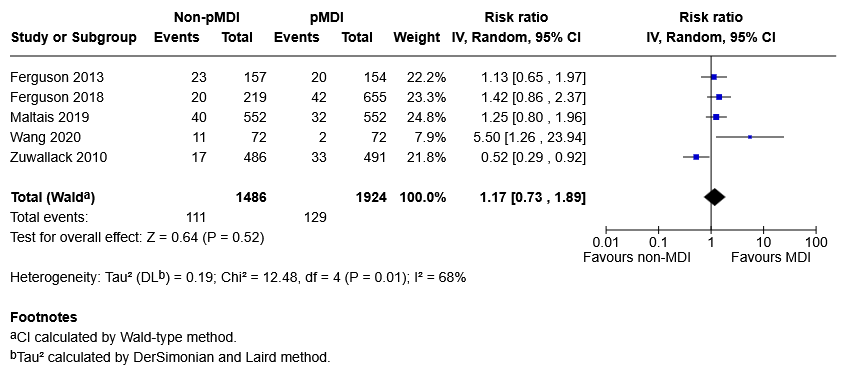


### Figure S10. Treatment-related adverse events (risk of >1) in (A) Asthma maintenance and (B) COPD

**A**


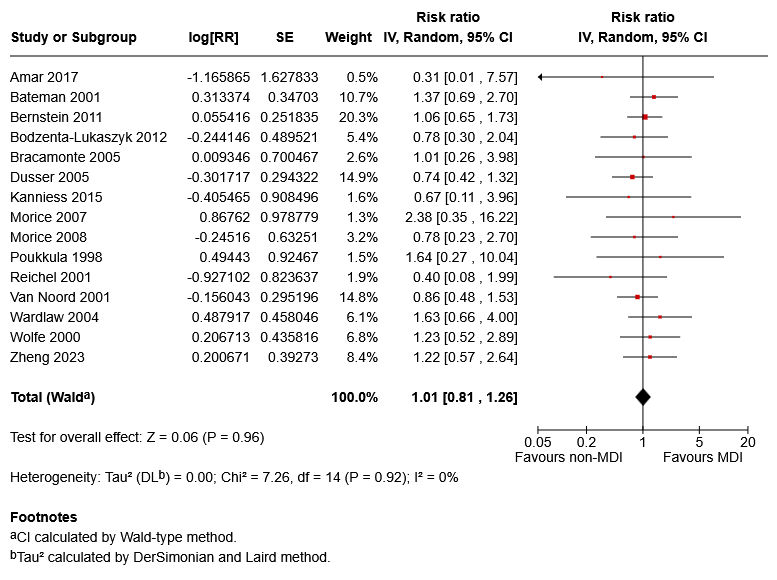


N = 5744

**B**


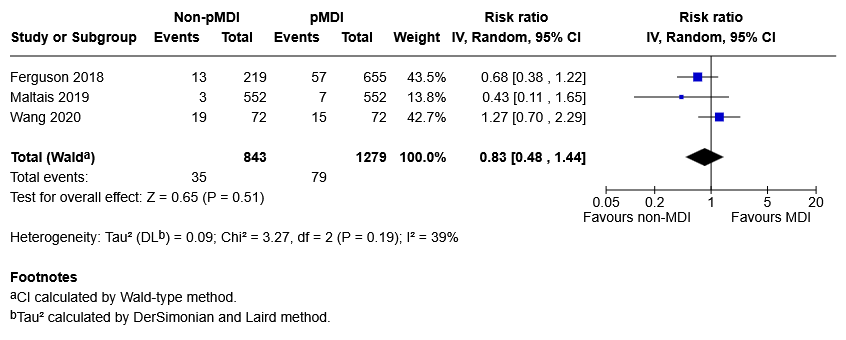


### Figure S11. Treatment-related serious adverse events (risk of >1) in (A) Asthma maintenance and (B) COPD

**A**


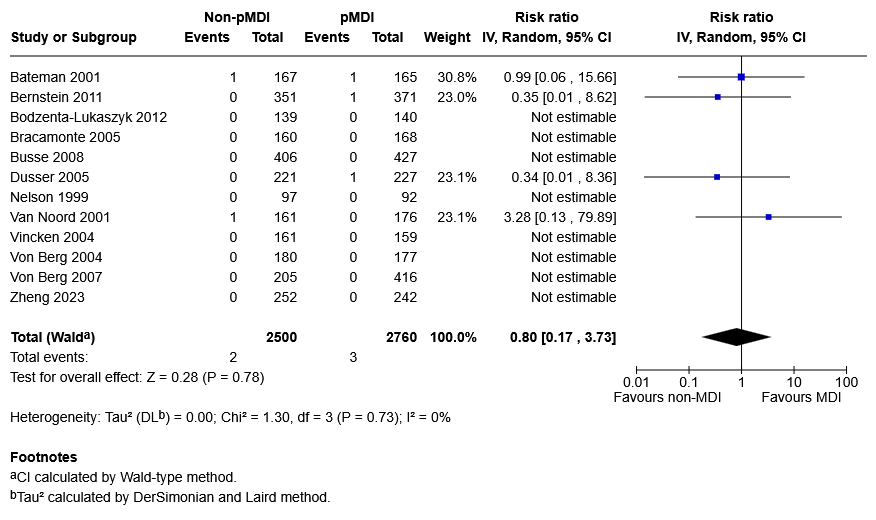
**B**


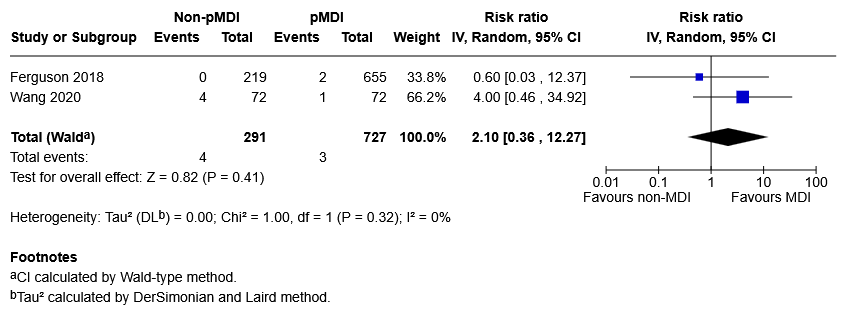


## Mortality

### Figure S12. Mortality in COPD


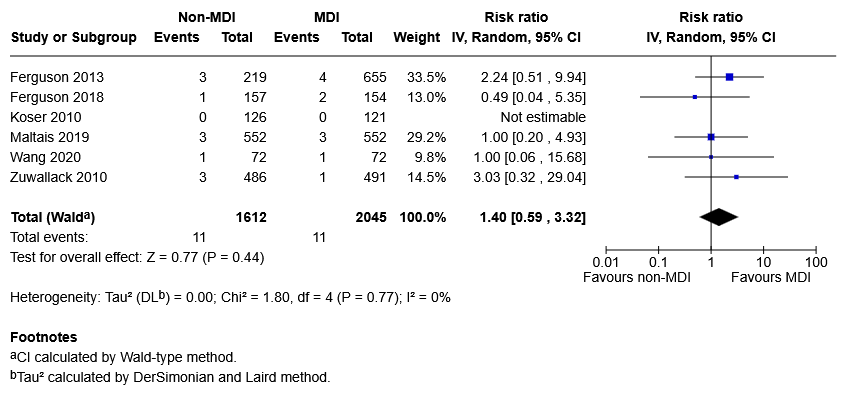


## Subgroup analyses

### Figure S13. Subgroup analysis of FEV1 by age of participants in Asthma Maintenance


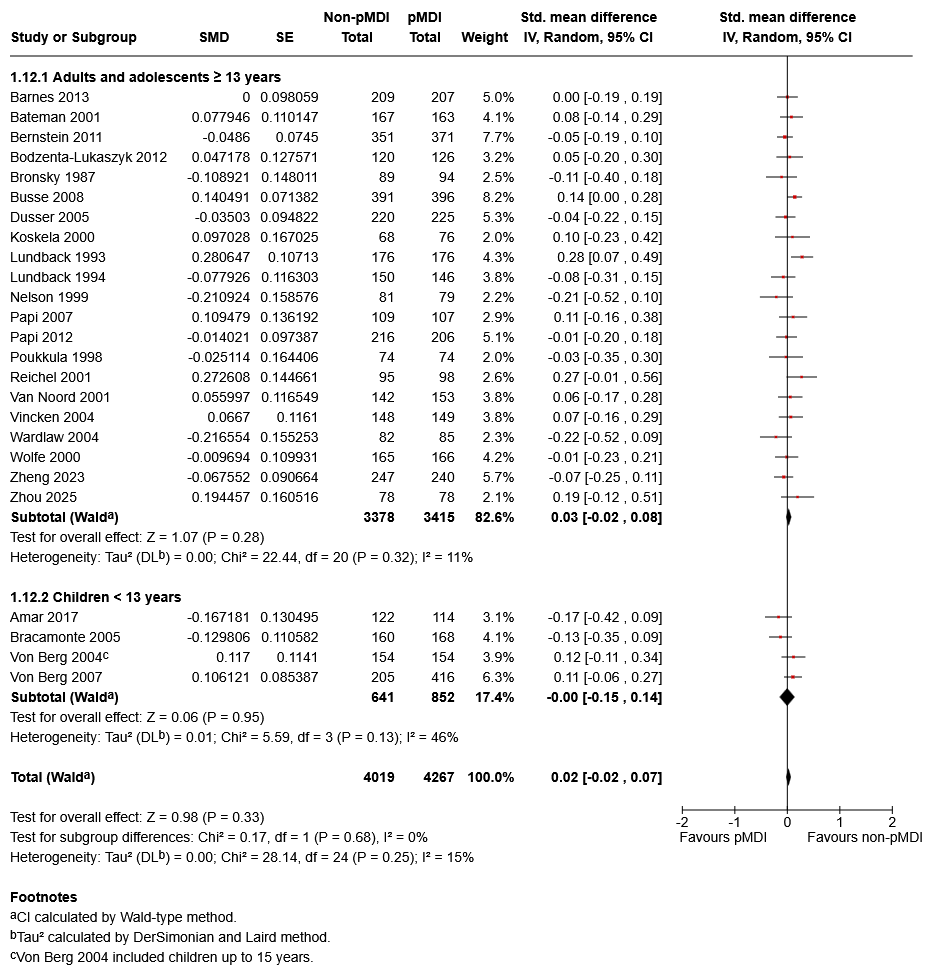


### Figure S14. Subgroup analysis of FEV1 by non-pMDI device type in COPD


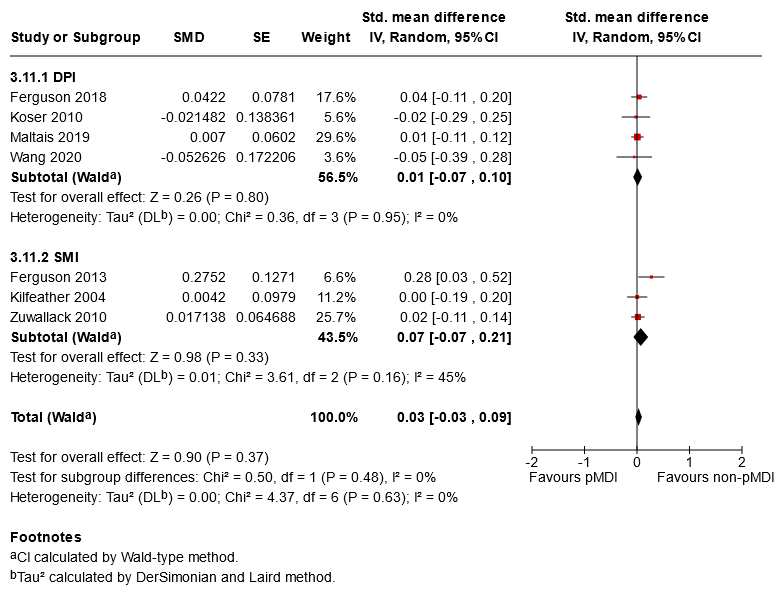


N = 1624

N = 2322

N = 3946

### Figure S15. Subgroup analysis of FEV1 by manufacturer funding in Asthma Maintenance


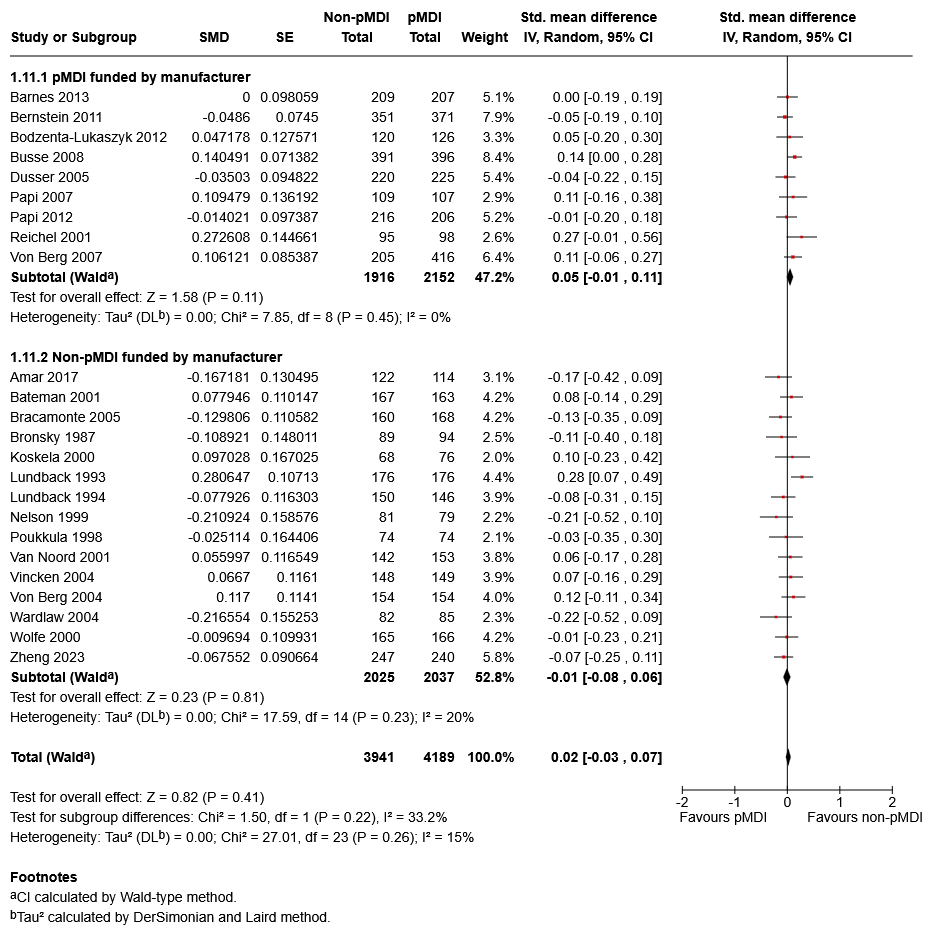


# Section E. Additional studies

## Table S15 – Excluded studies that might appear to meet inclusion criteria

| **Number** | **Citation** | **Reason for exclusion** |
| --- | --- | --- |
| 1 | Ali Rizvi, D., M. Tariq Salman, J. Sircar and A. Ahmad (2013). "Effect of Budesonide by metered dose inhaler with or without spacer & dry powder inhaler on Lung Function." International Journal of Drug Development and Research **5**(4): 233-240. | Wrong outcomes |
| 2 | Bensch, G., R. J. Lapidus, B. E. Levine, W. Lumry, U. Yegen, P. Kiselev and G. Della Cioppa (2001). "A randomized, 12-week, double-blind, placebo-controlled study comparing formoterol dry powder inhaler with albuterol metered-dose inhaler." Ann Allergy Asthma Immunol **86**(1): 19-27. | Wrong comparator |
| 3 | Berger, R. and W. E. Berger (2013). "Particle size and small airway effects of mometasone furoate delivered by dry powder inhaler." Allergy Asthma Proc **34**(1): 52-58. | Wrong outcomes |
| 4 | Bogdan, M. A., H. Aizawa, Y. Fukuchi, M. Mishima, M. Nishimura and M. Ichinose (2011). "Efficacy and safety of inhaled formoterol 4.5 and 9 mug twice daily in Japanese and European COPD patients: Phase III study results." BMC Pulmonary Medicine **11**: 51. | Wrong comparator |
| 5 | Bulac, S., A. Cimrin and H. Ellidokuz (2015). "The effect of beclometasone dipropionate/formoterol extra-fine fixed combination on the peripheral airway inflammation in controlled asthma." J Aerosol Med Pulm Drug Deliv **28**(2): 82-87. | Wrong outcomes |
| 6 | Calverley, P. M., P. Kuna, E. Monsó, M. Costantini, S. Petruzzelli, F. Sergio, G. Varoli, A. Papi and V. Brusasco (2010). "Beclomethasone/formoterol in the management of COPD: a randomised controlled trial." Respir Med **104**(12): 1858-1868. | Wrong dose |
| 7 | Capanoglu, M., E. Dibek Misirlioglu, M. Toyran, E. Civelek and C. N. Kocabas (2015). "Evaluation of inhaler technique, adherence to therapy and their effect on disease control among children with asthma using metered dose or dry powder inhalers." J Asthma **52**(8): 838-845. | Wrong outcomes |
| 8 | Chang, T. Y., J. Y. Chien, C. H. Wu, Y. H. Dong and F. J. Lin (2019). "Comparative Safety and Effectiveness of Inhaled Corticosteroids and Long-Acting beta2 Agonist Combinations in Patients with Chronic Obstructive Pulmonary Disease." Chest **157**(5): 1117-1129. | Wrong comparator |
| 9 | Chapman, K. R., K. Friberg, M. S. Balter, R. H. Hyland, M. Alexander, R. T. Abboud, S. Peters and B. H. Jennings (1997). "Albuterol via Turbuhaler versus albuterol via pressurized metered-dose inhaler in asthma." Ann Allergy Asthma Immunol **78**(1): 59-63. | Wrong outcomes |
| 10 | Chuchalin, A. G., H. J. Kremer, P. Metzenauer, E. O'Keefe and R. Hermann (2002). "Clinical equivalence trial on budesonide delivered either by the Novolizer multidose dry powder inhaler or the Turbuhaler in asthmatic patients." Respiration **69**(6): 502-508. | Wrong comparator |
| 11 | Corren, J., P. E. Korenblat, C. J. Miller, C. D. O'Brien and W. S. Mezzanotte (2007). "Twelve-week, randomized, placebo-controlled, multicenter study of the efficacy and tolerability of budesonide and formoterol in one metered-dose inhaler compared with budesonide alone and formoterol alone in adolescents and adults with asthma." Clin Ther **29**(5): 823-843. | Wrong comparator |
| 12 | Crompton, G. K., R. Sanderson, M. H. Dewar, S. P. Matusiewicz, A. C. Ning, A. H. Jamieson, A. McLean and A. P. Greening (2000). "Comparison of Pulmicort pMDI plus Nebuhaler and Pulmicort Turbuhaler in asthmatic patients with dysphonia." Respir Med **94**(5): 448-453. | Wrong outcomes |
| 13 | Cuvelier, A., J. F. Muir, D. Benhamou, E. Weitzenblum, P. Zuck, R. Delacenserie, A. Taytard and P. Iacono (2002). "Dry powder ipratropium bromide is as safe and effective as metered-dose inhaler formulation: a cumulative dose-response study in chronic obstructive pulmonary disease patients." Respir Care **47**(2): 159-166. | Wrong outcomes |
| 14 | Du, Y., W. Wang, W. Yang and B. He (2014). "Interleukin-32, not reduced by salmeterol/fluticasone propionate in smokers with chronic obstructive pulmonary disease." Chin Med J (Engl) **127**(9): 1613-1618. | Wrong outcomes |
| 15 | Ferguson, G. T., N. Brown, C. Compton, T. C. Corbridge, K. Dorais, C. Fogarty, C. Harvey, M. C. Kaisermann, D. A. Lipson, N. Martin, F. Sciurba, M. Stiegler, C. Q. Zhu and D. Bernstein (2020). "Once-daily single-inhaler versus twice-daily multiple-inhaler triple therapy in patients with COPD: lung function and health status results from two replicate randomized controlled trials." Respir Res **21**(1): 131. | Wrong comparator |
| 16 | Giraud, V. and F. A. Allaert (2009). "Improved asthma control with breath-actuated pressurized metered dose inhaler (pMDI): the SYSTER survey." Eur Rev Med Pharmacol Sci **13**(5): 323-330. | Wrong comparator |
| 17 | Hatter L, Holliday M, Eathorne A, Bruce P, Pavord ID, Reddel HK, Hancox RJ, Papi A, Weatherall M, Beasley R. The carbon footprint of as-needed budesonide/formoterol in mild asthma: a post hoc analysis. Eur Respir J. 2024 Jul 11;64(1):2301705. | Wrong comparator |
| 18 | Hirsch, T., M. Peter-Kern, R. Koch and W. Leupold (1997). "Influence of inspiratory capacity on bronchodilatation via Turbuhaler or pressurized metered-dose inhaler in asthmatic children: a comparison." Respir Med **91**(6): 341-346. | Wrong outcomes |
| 19 | Horiguchi, T., N. Hayashi, D. Ohira, H. Torigoe, T. Ito, M. Hirose, Y. Sasaki, M. Shiga, J. Miyazaki, R. Kondo and S. Tachikawa (2006). "Usefulness of HFA-BDP for adult patients with bronchial asthma: randomized crossover study with fluticasone." J Asthma **43**(7): 509-512. | Wrong comparator |
| 20 | Huber, B., C. Keller, M. Jenkins, A. Raza and M. Aurivillius (2022). "Effect of inhaled budesonide/formoterol fumarate dihydrate delivered via two different devices on lung function in patients with COPD and low peak inspiratory flow." Ther Adv Respir Dis **16**: 17534666221107312. | Wrong outcomes |
| 21 | Ige, O. M. and O. M. Sogaolu (2004). "A single blinded randomised trial to compare the efficacy and safety of once daily budesonide (400microg) administered by turbuhaler with beclomethasone dipropionate (400microg) given twice daily through a metered-dose inhaler in patients with mild to moderate asthma." Afr J Med Med Sci **33**(2): 155-160. | Wrong dose |
| 22 | Kawai, M., A. Sakai, S. Takaori, A. Hiura, N. Sakata, M. Nakashima and T. Miyamoto (2005). "Pharmacodynamic study of procaterol hydrochloride dry powder inhaler: evaluation of pharmacodynamic equivalence between procaterol hydrochloride dry powder inhaler and procaterol hydrochloride metered-dose inhaler in asthma patients in a randomized, double-dummy, double-blind crossover manner." Methods Find Exp Clin Pharmacol **27**(6): 385-389. | Wrong outcomes |
| 23 | Karjalainen J, Vartiainen V, Tikkakoski A, Malmberg LP, Vuotari L, Lähelmä S, Sairanen U, Vahteristo M, Lehtimäki L. Salbutamol Easyhaler provides non-inferior relief of methacholine induced bronchoconstriction in comparison to Ventoline Evohaler with spacer: A randomized trial. Respir Med. 2024 Aug-Sep;230:107693 | Wrong outcomes |
| 24 | Kerwin, E., A. Wachtel, L. Sher, J. Nyberg, P. Darken, S. Siddiqui, E. A. Duncan, C. Reisner and P. Dorinsky (2018). "Efficacy, safety, and dose response of glycopyrronium administered by metered dose inhaler using co-suspension delivery technology in subjects with intermittent or mild-to-moderate persistent asthma: A randomized controlled trial." Respir Med **139**: 39-47. | Wrong comparator |
| 25 | Khan A, Khan SA, Shah SMA, Sadiq F, Khaliq A, Nisar S. Comparison of Pulmonary Index Score after Treatment with Salbutamol Through Nebulisers <em>Vs.</em> Metered-Dose Inhalers with Spacer Device for Treatment of Childhood Wheeze. J Coll Physicians Surg Pak. 2025 Jul;35(7):932-934. | Wrong comparator |
| 26 | Koh HP, Lai SN, Chong WW, Mohd Pauzi Z. Budesonide/formoterol turbuhaler vs pMDI salbutamol for acute asthma in outpatient emergency department: a prospective, randomized, open-label study. J Asthma. 2025 Apr;62(4):694-704. | Wrong comparator |
| 27 | Kolasani, B. P., V. M. Lanke and S. Diyya (2013). "Influence of delivery devices on efficacy of inhaled fluticasone propionate: a comparative study in stable asthma patients." J Clin Diagn Res **7**(9): 1908-1912. | Wrong outcomes |
| 28 | Kostikas, K., J. F. Maspero, K. R. Chapman, K. Mezzi, X. Jaumont, D. Lawrence and R. van Zyl-Smit (2023). "Efficacy of mometasone/indacaterol/glycopyrronium in patients with inadequately controlled asthma with respect to baseline eosinophil count: Post hoc analysis of IRIDIUM study." Respiratory Medicine **217**: 107334. | Wrong comparator |
| 29 | Kraszko, P., D. Vondra, J. Malolepszy, M. Svensson, J. Baly, J. Fiserova, J. Jirkal, K. Kalandrova, P. Pancner, I. Michl, E. Ohnutkova, L. Pavelkova, P. Petrik, T. Sykora, R. Vodrazka, D. Chvatalova, G. Berta, B. Gautier, G. B. Nagy, I. Tallosy, Z. Gonczi, G. Czerniawska-Mysik, T. Hofman, R. Sopel, M. Szmidt and M. L. Kowalski (1999). "Budesonide via Turbuhaler, 400 mug daily, is as effective as beclomethasone dipropionate via pressurised MDI, 800 mug daily, for control of mild to moderate asthmatic patients." Journal of Clinical Research **2**(47-55): 47-55. | Wrong dose |
| 30 | Kupczyk, M., P. Majak, P. Kuna, B. Asankowicz-Bargiel, E. Barańska, R. Dobek, S. Garbicz, J. Jerzyńska, A. Latos, W. Machowiak, B. Majorek-Olechowska, A. Olech-Cudzik, I. Poziomkowska-Gęsicka, M. Rulewicz-Warniełło, A. Świderska, M. Tarnowski and P. Kopyto (2021). "A new formulation of fluticasone propionate/salmeterol in a metered-dose inhaler (MDI HFA) allows for the reduction of a daily dose of corticosteroid and provides optimal asthma control - A randomized, multi-center, non-inferiority, phase IV clinical study." Respir Med **176**: 106274. | Wrong dose |
| 31 | Kupczyk M, Panek M, Sadafi H, De Backer W, Wojakiewicz M, Dębowski T. Lung Deposition of Extrafine Versus Nonextrafine Aerosols at Low Inhalation Flow Rates in Adult Asthma Patients: A Composition Study. J Aerosol Med Pulm Drug Deliv. 2025 Aug;38(4):202-210. | Wrong outcomes |
| 32 | LaForce, C., B. M. Prenner, K. Andriano, C. Lavecchia and U. Yegen (2005). "Efficacy and safety of formoterol delivered via a new multidose dry powder inhaler (Certihaler) in adolescents and adults with persistent asthma." J Asthma **42**(2): 101-106. | Wrong comparator |
| 33 | Lal, S., S. M. Malhotra, M. D. Gribben and A. G. Butler (1980). "Beclomethasone dipropionate aerosol compared with dry powder in the treatment of asthma." Clin Allergy **10**(3): 259-262. | Wrong comparator |
| 34 | Löfdahl, C. G., L. Andersson, E. Bondesson, L. G. Carlsson, K. Friberg, J. Hedner, Y. Hörnblad, P. Jemsby, A. Källén, A. Ullman, S. Werner and N. Svedmyr (1997). "Differences in bronchodilating potency of salbutamol in Turbuhaler as compared with a pressurized metered-dose inhaler formulation in patients with reversible airway obstruction." Eur Respir J **10**(11): 2474-2478. | Wrong outcomes |
| 35 | McCarthy, P., T. Iliadis and K. Zaiken (2022). "Clinical Response and Cost-Savings Associated With Generic Fluticasone Propionate/Salmeterol Multidose, Dry-Powder Inhaler in Asthma Patients Managed in an Ambulatory Care Practice Setting." J Pharm Pract **35**(2): 274-280. | Wrong comparator |
| 36 | Miyamoto, T., T. Takahashi, S. Nakajima, S. Makino, M. Yamakido, K. Mano, M. Nakashima, U. Tollemar and O. Selroos (2001). "Efficacy of budesonide Turbuhaler compared with that of beclomethasone dipropionate pMDI in Japanese patients with moderately persistent asthma." Respirology **6**(1): 27-35. | Wrong dose |
| 37 | Ng DX, Leow MQH, Koh YLE, Aau WK, Tan NC. Resultant greenhouse gases from the use of inhaled corticosteroid based on Global Initiative for Asthma (GINA) guidelines: a primary care used case from Singapore. NPJ Prim Care Respir Med. 2025 Jul 26;35(1):35. | Wrong comparator |
| 38 | Noonan, M., L. J. Rosenwasser, P. Martin, C. D. O'Brien and L. O'Dowd (2006). "Efficacy and safety of budesonide and formoterol in one pressurised metered-dose inhaler in adults and adolescents with moderate to severe asthma: a randomised clinical trial." Drugs **66**(17): 2235-2254. | Wrong comparator |
| 39 | O'Callaghan, C., M. L. Everard, A. Bush, E. J. Hiller, R. Ross-Russell, P. O'Keefe and P. Weller (2002). "Salbutamol dry powder inhaler: efficacy, tolerability, and acceptability study." Pediatr Pulmonol **33**(3): 189-193. | Wrong outcomes |
| 40 | Ohaju-Obodo, J. O., C. Chukwu, J. Okpapi, E. Egbagbe, M. O. Ige, C. Chukwuka and D. A. Adedapo (2005). "Comparison of the efficacy and safety of budesonide turbuhaler administered once daily with twice the dose of beclomethasone dipropionate using pressurised metered dose inhaler in patients with mild to moderate asthma." West Afr J Med **24**(3): 190-195. | Wrong dose |
| 41 | Ohbayashi, H., S. Kudo and M. Ariga (2018). "Evaluation of Rapid Onset of Action of ICS/LABA Combination Therapies on Respiratory Function in Asthma Patients: A Single-Center, Open-Label, Randomized, Crossover Trial." Pulm Ther **4**(2): 159-169. | Wrong outcomes |
| 42 | Onasanya AA, Haider Y, Peaston G, Ignatowicz A, Turner AM. Inhaler sustainability in asthma and COPD care: a systematic review. BMJ Open. 2025 Jul 25;15(7):e098052. | Wrong outcomes |
| 43 | Piquet, J., P. Zuck, G. Dennewald, P. Dugue, M. Grivaux, P. Brun, J. C. Severac, J. Ostinelli and K. H. Cheeseman (1996). "Equally efficacious asthma management with budesonide 800 micrograms administered by Turbuhaler or with beclomethasone dipropionate > or = 1500 micrograms given through a pressurized metered-dose inhaler with spacer. The French Budesonide Trial Group." Adv Ther **13**(1): 38-50. | Wrong dose |
| 44 | Price, D., V. Thomas, J. von Ziegenweidt, S. Gould, C. Hutton and C. King (2014). "Switching patients from other inhaled corticosteroid devices to the Easyhaler(®): historical, matched-cohort study of real-life asthma patients." J Asthma Allergy **7**: 31-51. | Wrong comparator |
| 45 | Razzouk, H., L. dos Santos, J. Giudicelli, M. Queirós, M. de Lurdes Chieira, A. Castro, C. Ramos and C. Lindbladh (1999). "A comparison of the bronchodilatory effect of 50 and 100 microg salbutamol via Turbuhaler and 100 microg salbutamol via pressurized metered dose inhaler in children with stable asthma." Int J Pharm **180**(2): 169-175. | Wrong outcomes |
| 46 | Rodrigo, G. J., H. Neffen, F. D. Colodenco and J. A. Castro-Rodriguez (2010). "Formoterol for acute asthma in the emergency department: a systematic review with meta-analysis." Ann Allergy Asthma Immunol **104**(3): 247-252. | Wrong comparator |
| 47 | Salvi, S., A. K. Deb, M. Agarwal, V. R. Tummuru, R. Kodgule, V. S. Hemalatha, A. K. Awasthi, K. P. Suraj, V. K. Pavitran, S. P. Mourya, P. Thomas, A. Vaidya, S. Chhowala and J. Gogtay (2020). "Fixed-dose combination of three drugs, i.e. LABA/LAMA/ICS for COPD: Results of a real-world study from India." Pulm Pharmacol Ther **63**: 101932. | Wrong comparator |
| 48 | Schurmann, W., S. Schmidtmann, P. Moroni, D. Massey and M. Qidan (2005). "Respimat Soft MistTM inhaler versus hydrofluoroalkane metered dose inhaler: Patient preference and satisfaction." Treatments in Respiratory Medicine **4**(1): 53-61. | Wrong dose |
| 49 | Selroos, O., R. Backman, K. O. Forsen, A. B. Lofroos, M. Niemisto, A. Pietinalho and H. Riska (1994). "Clinical efficacy of budesonide Turbuhalerregistered trade mark compared with that of beclomethasone dipropionate pMDI with volumatic spacer. A 2-year randomized study in 102 asthma patients." ALLERGY-EUR-J-ALLERGY-CLIN-IMMUNOL **49**(10): 833‐836. | Wrong comparator |
| 50 | Singh, D., G. Nicolini, E. Bindi, M. Corradi, D. Guastalla, J. Kampschulte, W. Pierzchala, A. Sayiner, M. Szilasi, C. Terzano and J. Vestbo (2014). "Extrafine beclomethasone/formoterol compared to fluticasone/salmeterol combination therapy in COPD." BMC Pulmonary Medicine **14**(1): 43. | Wrong dose |
| 51 | Singh D, Bafadhel M, Arya N, Marshall J, Parikh H, Kisielewicz D, Movitz C, Bowen K, Patel M. Step up to triple therapy versus switch to dual bronchodilator therapy in patients with COPD on an inhaled corticosteroid/long-acting β2-agonist: post-hoc analyses of KRONOS. Respir Res. 2025 May 8;26(1):175. | Wrong comparator |
| 52 | Tamási, L., M. Szilasi and G. Gálffy (2018). "Clinical Effectiveness of Budesonide/Formoterol Fumarate Easyhaler(®) for Patients with Poorly Controlled Obstructive Airway Disease: a Real-World Study of Patient-Reported Outcomes." Adv Ther **35**(8): 1140-1152. | Wrong comparator |
| 53 | Tammivaara, R., E. Aalto, K. Lehtonen, V. Vilkka, K. Laurikainen, M. Silvasti, P. Toivanen and H. Tukiainen (1997). "Comparison of a novel salbutamol multidose powder inhaler with a salbutamol metered dose inhaler in patients with asthma." Current Therapeutic Research - Clinical and Experimental **58**(10): 734-744. | Wrong comparator |
| 54 | Usmani OS, Martinez FJ, Pandya H, Camiolo M, Bednarczyk A, Kucz K, Kokot M, Gottfridsson C, Aurivillius M, Pettersson L, Mei J, Skansen K, Bell JL, Petullo D, Collison K, Bondarov P, Jassal M, Patel M. Safety of budesonide/glycopyrronium/formoterol fumarate dihydrate delivered by HFO-1234ze versus HFA-134a in chronic obstructive pulmonary disease: a phase 3, multi-site, randomised, double-blind, parallel-group, active-comparator study. EClinicalMedicine. 2025 Aug 12;87:103402. | Wrong comparator |
| 55 | van den Bosch WB, Ruijgrok EJ, Tousi NM, Tiddens HAWM, Janssens HM. Small Airways Disease Affects Aerosol Deposition in Children with Severe Asthma: A Functional Respiratory Imaging Study. J Aerosol Med Pulm Drug Deliv. 2024 Dec;37(6):351-361. doi: 10.1089/jamp.2024.0005. Epub 2024 Sep 4. | Wrong outcomes |
| 56 | van Geffen, W. H., W. R. Douma, D. J. Slebos and H. A. Kerstjens (2016). "Bronchodilators delivered by nebuliser versus pMDI with spacer or DPI for exacerbations of COPD." Cochrane Database Syst Rev **2016**(8): Cd011826. | Wrong comparator |
| 57 | Vidgren, P., M. Silvasti, A. Poukkula, K. Laasonen and M. Vidgren (1994). "Easyhaler powder inhaler - A new alternative in the anti-inflammatory treatment of asthma." Acta Therapeutica **20**(3-4): 117-131. | Wrong outcomes |
| 58 | Vinge, I., J. Syk, A. Xanthopoulos, H. Laßmann, M. Vahteristo, U. Sairanen, S. Lähelmä, R. Hennig and M. Müller (2021). "A non-interventional switch study in adult patients with asthma or COPD on clinical effectiveness of salmeterol/fluticasone Easyhaler(®) in routine clinical practice." Ther Adv Respir Dis **15**: 17534666211027787. | Wrong comparator |
| 59 | Vieira RJ, Duarte VH, Bognanni A, Gil-Mata S, Marques-Cruz M, Pereira AM, Chen-Xu J, Louis R, Williams S, Nadeau KC, Haahtela T, Piggott T, Fonseca JA, Antó JM, Schünemann HJ, Bousquet J, Sousa-Pinto B. Comparison of Three Treatment Strategies in Mild Asthma: A Carbon-Utility Analysis. J Allergy Clin Immunol Pract. 2025 Jul 7:S2213-2198(25)00627-0. doi: 10.1016/j.jaip.2025.06.035. Epub ahead of print | Wrong comparator |
| 60 | Voshaar, T., R. Lapidus, R. Maleki-Yazdi, W. Timmer, E. Rubin, L. Lowe and E. Bateman (2008). "A randomized study of tiotropium Respimat Soft Mist inhaler vs. ipratropium pMDI in COPD." Respiratory Medicine **102**(1): 32‐41. | Wrong comparator |
| 61 | Woodcock, A., C. Janson, J. Rees, L. Frith, M. Löfdahl, A. Moore, M. Hedberg and D. Leather (2022). "Effects of switching from a metered dose inhaler to a dry powder inhaler on climate emissions and asthma control: post-hoc analysis." Thorax **77**(12): 1187-1192. | Wrong dose |
| 62 | Worth, H., J. F. Muir and W. R. Pieters (2001). "Comparison of hydrofluoroalkane-beclomethasone dipropionate AutohalerTM with budesonide TurbuhalerTM in asthma control." Respiration **68**(5): 517-526. | Wrong dose |
| 63 | Zheng, J. P., L. Yang, Y. M. Wu, P. Chen, Z. G. Wen, W. J. Huang, Y. Shi, C. Z. Wang, S. G. Huang, T. Y. Sun, G. F. Wang, S. D. Xiong and N. S. Zhong (2007). "The efficacy and safety of combination salmeterol (50 microg)/fluticasone propionate (500 microg) inhalation twice daily via accuhaler in Chinese patients with COPD." Chest **132**(6): 1756-1763. | Wrong comparator |
